# Chromosome-Scale Reference Genome of *Amphicarpaea edgeworthii*: A New Resource for Amphicarpic Plants Research and Complex Flowering Pattern

**DOI:** 10.3389/fpls.2021.770660

**Published:** 2021-11-18

**Authors:** Tingting Song, Mengyan Zhou, Yuying Yuan, Jinqiu Yu, Hua Cai, Jiawei Li, Yajun Chen, Yan Bai, Gang Zhou, Guowen Cui

**Affiliations:** ^1^Department of Grassland Science, College of Animal Science and Technology, Northeast Agricultural University, Harbin, China; ^2^Novogene Bioinformatics Institute, Beijing, China

**Keywords:** *Amphicarpaea edgeworthii*, amphicarpic plant, comparative genomics, genome evolution, flower and seed development

## Abstract

*Amphicarpaea edgeworthii*, an annual twining herb, is a widely distributed species and an attractive model for studying complex flowering types and evolutionary mechanisms of species. Herein, we have generated a high-quality assembly of *A. edgeworthii* by using a combination of PacBio, 10× Genomics libraries, and Hi-C mapping technologies. The final 11 chromosome-level scaffolds covered 90.61% of the estimated genome (343.78Mb), which is a chromosome-scale assembled genome of an amphicarpic plant. Subsequently, we characterized the genetic diversity and population structure of *A. edgeworthii* species by resequencing individuals collected from their natural area of distribution. Using transcriptome profiling, we observed that specific phenotypes are regulated by a complex network of light, hormones, and MADS-box gene families. These data are beneficial for the discovery of genes that control major agronomic traits and spur genetic improvement of and functional genetic studies in legumes, as well as supply comparative genetic resources for other amphicarpic plants.

## Introduction

In nature, the distribution of key resources required for plant growth is often uneven. Plants growing in unstable habitats, with limited supplies of mineral nutrients, water, or light, frequent soil interferences, and large environmental fluctuations, undergo adaptive evolution to improve their survival (Jackson and Caldwell, 1993; Pearcy and Caldwell, 1994). Some plant species that bear two or more heteromorphic flowers also bear heteromorphic fruits (seeds). Amphicarpy is a phenomenon in which a plant produces both aerial and subterranean flowers and simultaneously bears both aerial and subterranean fruits on aerial and subterranean stems, respectively (Schnee and Waller, 1986; Cheplick, 1987; Koontz et al., 2017). This phenomenon is observed in at least 67 herbaceous species (31 in Fabaceae) in 39 genera and 13 families of angiosperms, as reported by Zhang et al. (2020a). Amphicarpy is an important part of plant adaptive evolution, in which angiosperms generally display a special type of fruiting pattern and different fruit (seed) types also exhibit various dormancy and morphological features. This type of fruiting mode is crucial for the ecological adaptation of plants because it reduces competition among siblings within the population, maintains and increases the population size *in situ*, and increases the adaptability and evolutionary plasticity of the species (Sadeh et al., 2009; Hidalgo et al., 2016).

**Figure 1:**
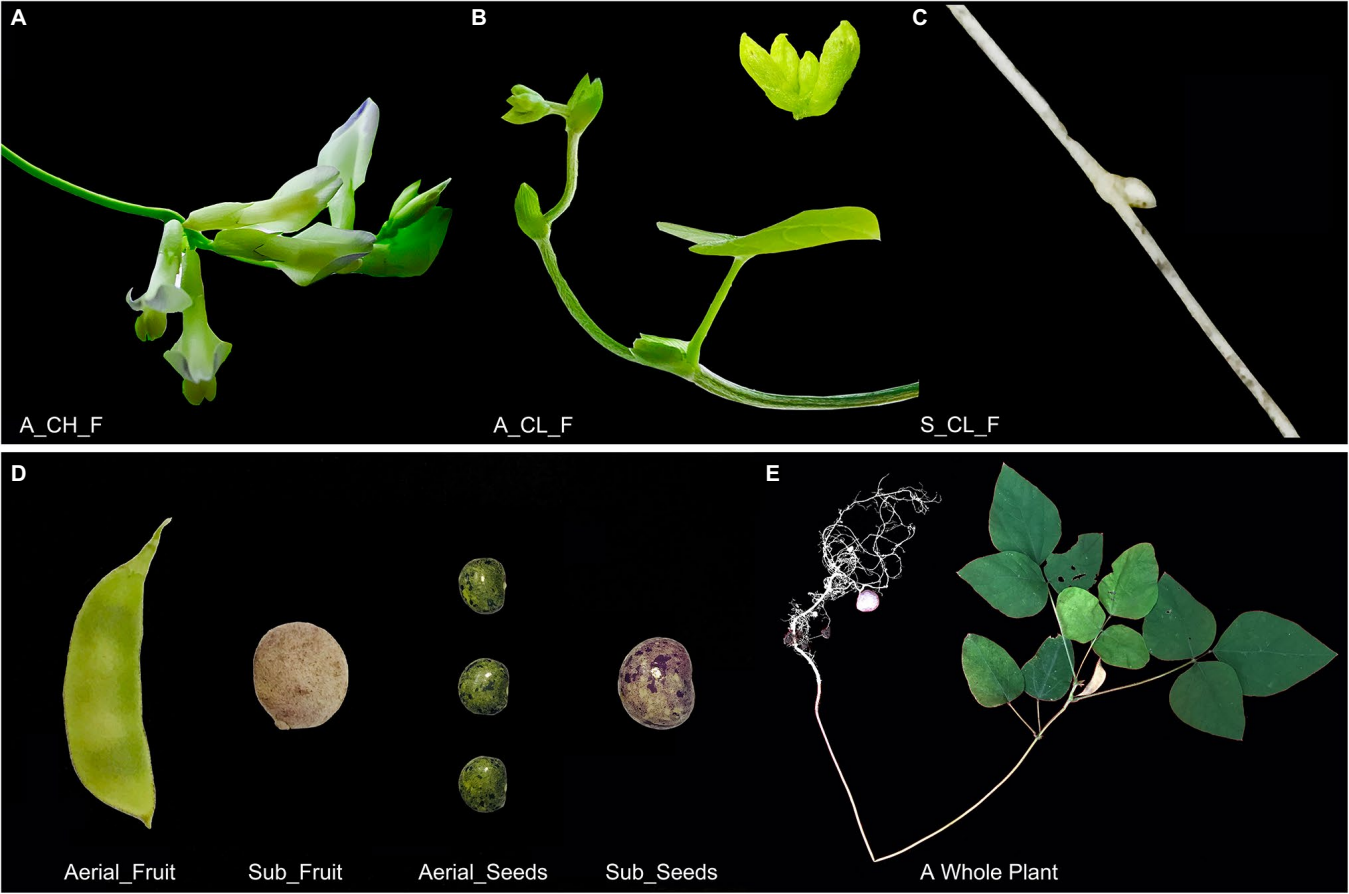
Morphological features of the *Amphicarpaea edgeworthii*. **(A)** Aerial chasmogamous flower (A_CH_F). **(B)** Aerial cleistogamous flower (A_CL_F). **(C)** Subterranean cleistogamous flower (S_CL_F). **(D)** Fruit and seed: Left is the aerial fruit/seed (both aerial flowers produce fruit with the same phenotype); right is the subterranean fruit/seed. **(E)** A whole plant of wild *A. edgeworthii* species.

*Amphicarpaea edgeworthii*, an annual twining herb, belongs to the Fabaceae, which is a large and economically valuable family of flowering plants (Zhang et al., 2006, 2017). In this plant species, three types of flowers (fruits), grow on a single plant (Figures 1A–E), namely aerial chasmogamous flowers (A_CH_F), aerial cleistogamous flowers (A_CL_F), and subterranean cleistogamous flowers (S_CL_F); aerial chasmogamous flowers are only produced during summer (Zhang et al., 2005; Sadeh et al., 2009). This species offers an attractive model for examining gene regulatory networks that control chasmogamous and cleistogamous flowering in plants. However, the mechanism of flower development in amphicarpic plants, particularly in legumes, is sparsely understood. The present study was an attempt to enhance our understanding on the reproductive biology and the precise evolutionary mechanism in amphicarpic plants. We performed whole-genome sequencing of *A. edgeworthii*, to gain insights into the complex flowering pattern and evolutionary status. This reference genome represents an important foundation for further understanding of agronomics and molecular breeding in *A. edgeworthii*.

**Figure 2:**
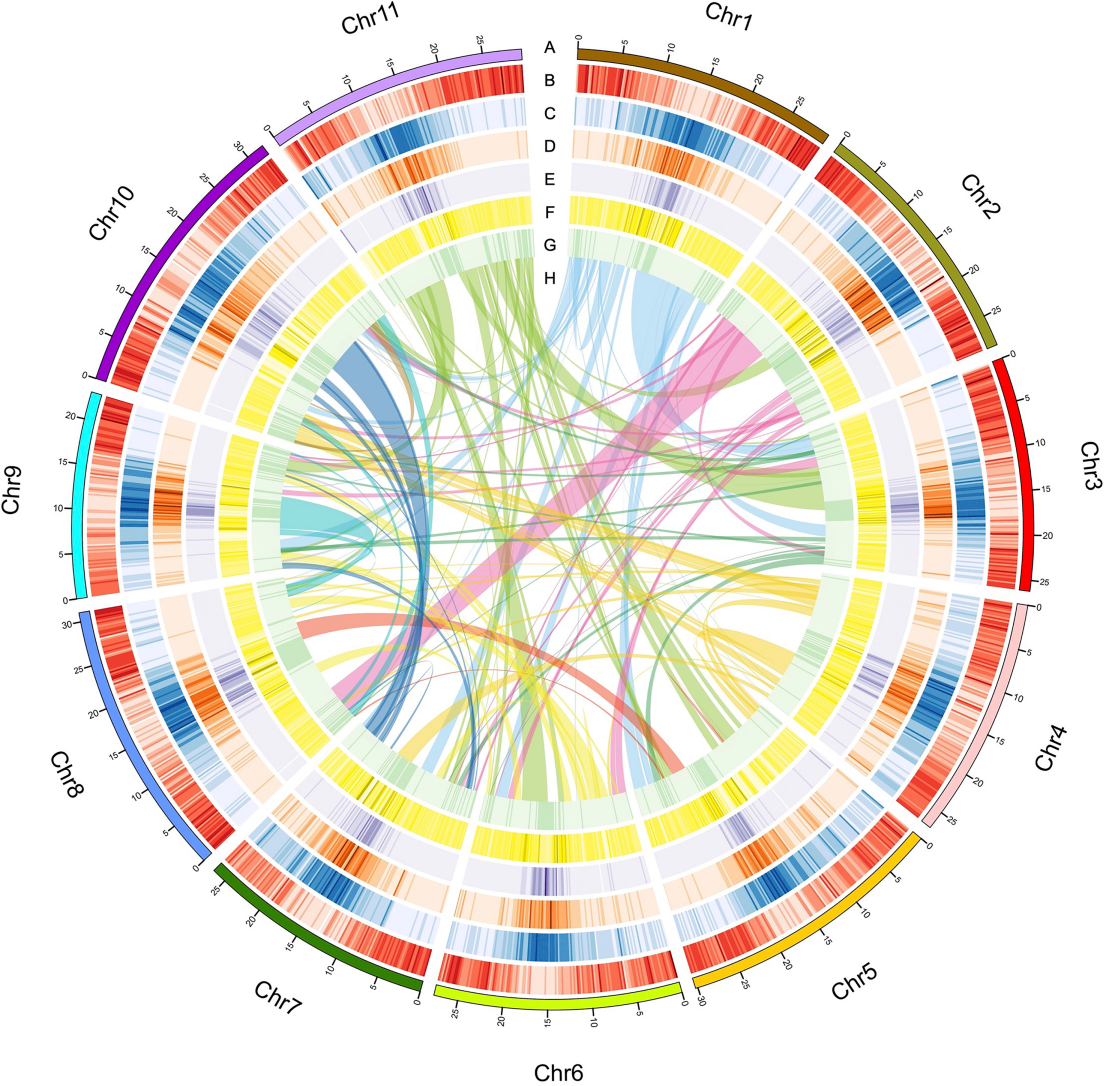
Characteristics of the *A. edgeworthii* genome. **(A)** Chromosome length; **(B)** Gene density per chromosome; **(C)** Repeat density; **(D)** LTR_Copia density; **(E)** LTR_Gypsy density; **(F)** SNP density in 5 populations; **(G)** Distribution of the GC content; **(H)** Intra-genome collinear blocks connected. All statistics are computed for windows of 200kb.

To this end, we leveraged PacBio long-read sequencing with the high-throughput chromosome conformation capture (Hi-C) technology to generate a chromosome-level genome assembly for *A. edgeworthii* (Figure 2; Supplementary Tables 1 and 2), which was used as a reference for the population genomics study of 48 individuals collected from 5 different regions of the species distribution. In addition, we performed comparative genomics to assess the phylogenetic relationships of the species with other legumes and angiosperms and compared the transcriptome data between different organs (flowers and seeds) to identify changes in gene expression with flower (seed) development. Our research findings could serve as a novel and valuable resources for research on amphicarpic plant biology and legume breeding.

## Materials and Methods

### Plant Material and Genome Survey

For genome sequencing, we collected fresh young leaves of *A. edgeworthii* species distributed in Heilongjiang Province (45.80°N, 126.53°E), China. The karyotype analysis of the plant species revealed a karyotype of 2n=2x=22, with uniform and small chromosomes (Wolny et al., 2013; Supplementary Figure 1).

We extracted DNA from the fresh leaves of *A. edgeworthii* by using a DNAsecure Plant Kit (TIANGEN, Biotech, China) and then purified and concentrated the isolated DNA; high-quality DNA was broken into random fragments, and Illumina paired-end library with 350-bp size was constructed and was sequenced using a Illumina HiSeq X-ten platform.

To estimate the *A. edgeworthii* genome size, high-quality short-insert reads (350-bp size) were used to extract the 17-mer sequences by using sliding windows. The frequency of each 17-mer was calculated and is presented in Supplementary Figure 2. Genome size was calculated by using the following formula:


Genome size=totalk−mernumbers/k−merdepth

The revised genome size was calculated after excluding the erroneous k-mers (Supplementary Table 1).

### Library Construction, Genome Sequencing, Assembly, and Evaluation

To construct long-insert libraries, we constructed SMRTbell libraries by following the standard protocol, as recommended by the manufacturer (PacBio Biosciences). Genomic DNA was broken into fragments of size 15kb–40kb, and large fragments were enriched, enzymatically repaired, and converted into SMRTbell libraries. SMRTbell libraries were sequenced using a PacBio Sequel platform.

The linked read sequencing libraries of 10× Genomics GemCode platform (Weisenfeld et al., 2017) were sequenced with 350-bp size by using an Illumina HiSeq X-ten platform.

Fresh leaves were plucked from the plant, and chromatin in the samples were crosslinked to DNA and fixed. A chromatin interaction mapping (Hi-C) library with 350-bp size was constructed for sequencing using Illumina HiSeq X-ten.

We used the FALCON software (Chin et al., 2016) for *de novo* assembly of PacBio SMRT reads (Supplementary Tables 2 and 3). Subreads with coverage higher than 60 were selected as seeds for assembly after pairwise comparisons of all the reads for error correction with default parameters. Error-corrected SMRT reads were aligned to each other to construct string graphs. After initial assembly, the produced contigs were polished using Quiver (Chin et al., 2013) with default parameters. The first round of error correction was performed using Illumina paired-end reads by Pilon (Walker et al., 2014). Subsequently, the scaffolding was performed using 10× Gscaff v.2.1 with 10× genomics data, and the genome was upgraded by PBjelly (English et al., 2012). The second round of error correction was performed using Illumina paired-end reads by Pilon (Walker et al., 2014). The Hi-C data were mapped to the original scaffold genome by using BWA v.0.7.7 (Li and Durbin, 2009), and only the reads with unique alignment positions were extracted to construct a chromosome-scale assembly by using the Ligating Adjacent Chromatin Enables Scaffolding In Situ (LACHESIS) tool (Burton et al., 2013; Supplementary Table 3).

### Genome Annotation

We used RepeatModeler, RepeatScout (Tarailo and Chen, 2009), Piler (Edgar and Myers, 2005), and LTR_FINDER (Xu and Wang, 2007) to develop a repeat library. RepeatMasker (Tarailo and Chen, 2009) was used for DNA-level identification in the Repbase and *de novo* transposable element libraries. Tandem repeats were ascertained in the genome by using Tandem Repeats Finder (Benson, 1999). RepeatProteinMask (Tarailo and Chen, 2009) was used to conduct WU-BLASTX searches against the transposable element protein database. Overlapping TEs belonging to the same type of repeats were integrated (Supplementary Tables 8 and 9).

To predict protein-coding genes in the *A. edgeworthii* genome, we used homolog-based prediction (using *Arachis duranensis*, *Cicer arietinum*, *Glycine max*, *Medicago truncatula*, *Phaseolus vulgaris*, and *Trifolium pratense* gene sets), *de novo* prediction [using Augustus v.3.0.2 (Stanke and Morgenstern, 2005), Genescan v.1.0 (Aggarwal and Ramaswamy, 2002), GeneID (Parra et al., 2000), GlimmerHMM v.3.0.2 (Majoros et al., 2004), SNAP (Korf, 2004) programs], and transcriptome-based prediction (using 5 tissue RNA sequencing data). A weighted and nonredundant gene set was generated using EVidenceModeler (EVM; Brian et al., 2008), which merged all the genes models, which were predicted using the aforementioned approaches. Along with the transcript assembly, the Program to Assemble Spliced Elements (Haas et al., 2003) was used to adjust the gene models generated using EVM (Supplementary Table 10).

Functional annotation of protein-coding genes was evaluated using BLASTP (E-value≤1E-05) against 2 integrated protein sequence databases, SwissProt (Bairoch and Apweiler, 2000) and NCBI nonredundant protein database. Protein domains were annotated by searching InterPro v.32.0, which included Pfam, PRINTS, PROSITE, ProDom, and SMART databases, by using InterProScan v.4.8 (Mulder and Apweiler, 2007). GO (Ashburner et al., 2000) terms for each gene were obtained from the corresponding InterPro descriptions. The pathways in which the gene might be involved were assigned using BLAST searches against the KEGG database (Kanehisa and Goto, 2000), with an E-value cutoff of 1E-05 (Supplementary Table 11).

The tRNA genes were predicted using tRNAscan-SE software (Lowe and Eddy, 1997). The miRNA and snRNA fragments were identified using INFERNAL software (Nawrocki et al., 2009) against the Rfam (Griffiths et al., 2005) database. The rRNA fragments were identified using BLASTN searches (E-value≤1E-10) against the plant rRNA database (Supplementary Table 12).

### Genome Evolution

To identify gene family clusters, nucleotide and protein data of 11 species (*Arabidopsis thaliana*, *A. duranensis*, *C. arietinum*, *G. max*, *Glycine soja*, *Glycyrrhiza uralensis*, *M. truncatula*, *P. vulgaris*, *Populus trichocarpa*, *Vigna angularis*, and *Vitis vinifera*) were downloaded from the Ensembl, NCBI, and JGI databases. Afterward, an “all against all” BLASTP program, with an E-value cutoff of 1E-07, was performed for proteins of 12 species, and the best BLAST hits were conjoined using Solar software (Yu et al., 2006). Finally, gene families were constructed using OrthoMCL (Li et al., 2003), with an inflation index of 1.5. Supplementary Tables 13 and 14 present the results of GO enrichment and KEGG enrichment analysis of unique gene families in *A. edgeworthii* species.

To reconstruct the phylogenetic tree of 12 species, protein data of shared single-copy orthologs were aligned using MUSCLE (Edgar, 2004) and the protein alignments were transformed to CDS alignments. We concatenated the CDS alignments of single-copy orthologs to a “supermatrix.” Using this supermatrix, we constructed the phylogenetic tree by using the ML (maximum-likelihood) TREE algorithm in RAxML software (Stamatakis, 2006). To estimate the divergence time, we applied MCMCtree program of PAML (Yang, 2007), with 3 fossils calibration points as prior settings, namely 100–120 Mya for the most recent common ancestor (TMRCA) of *A. thaliana*–*P. trichocarpa* (Tuskan et al., 2006); ≤60 Mya for TMRCA of *A. duranensis*–*G. max* (Lavin et al., 2005); and ≤125 Mya for TMRCA of *A. thaliana*–*V. vinifera* (Li et al., 2019).

We identified the expansion and contraction of the orthologous gene family by using the CAFE software (De Bie et al., 2006). To avoid extreme gene families, the families with gene number ≥200 in one species and ≤2 in all other species were filtered initially. Supplementary Tables 15 and 16 present the GO enrichment and KEGG enrichment analyses of expanded gene families in *A. edgeworthii*.

MCscan (Tang et al., 2008) was used to assess genome collinearity within *A. edgeworthii*, *G. max*, *G. soja*, *P. vulgaris*, and *V. angularis pretense*, respectively. We also assessed collinearity between *A. edgeworthii* and *G. max* and between *A. edgeworthii* and *G. soja*. Syntenic blocks containing at least 5 genes were obtained on the basis of similarity gene pairs generated using BLASTP searches, with an E-value cut-off of 1E-05. We extracted all the duplicated gene pairs from syntenic blocks and calculated the 4DTv distance.

### Genetic Structure and Introgression

Using the Illumina HiSeq X-ten platform, a total of 48 accessions, including HL (Heilongjiang), NM (Nei Mongol), SD (Shandong), HB (Hebei), and SC (Sichuan), were resequenced (Supplementary Table 19). Low-quality paired reads were excluded (Supplementary Table 20), and the remaining reads were mapped to the *A. edgeworthii* reference genome by using BWA (v.0.7.8). Duplicated reads were removed using SAMtools (v.1.3.1; Supplementary Table 20). SNP calling was performed on a population scale by using the “mpileup” command for SAMtools and the “call” command for BCFtools (v.1.3.1; Li et al., 2009). Only high-quality SNPs (coverage depth≥3, RMS mapping quality≥20, minor allele frequency≥0.05, and missing data≤0.1) were retained and annotated by ANNOVAR (v.2013-05-20; Wang et al., 2010; Supplementary Table 21) for subsequent analyses.

To assess the phylogenetic relationship of 48 accessions, an NJ tree was constructed using TreeBest software^1^ (v.1.9.2). To examine the genetic structure of the population based on an expectation maximization algorithm, we applied ADMIXTURE (v.1.23; Alexander et al., 2009), with the number of genetic clusters (K) ranging from 2 to 8. Principal components analysis (PCA) was conducted to evaluate the genetic structure by using software GCTA (Yang et al., 2011).

TreeMix (v.1.12; Pickrell and Pritchard, 2012) was used to deduce both the population splits and migration events. A maximum-likelihood phylogenetic tree was first constructed for the 5 populations of *A. edgeworthii*, with Nei Mongol population as the outgroup. Based on the genome-wide allelic frequency data, possible migration events were identified from a residual covariance matrix. We used parameters “-k 1,000” and “-m” ranging from 0 to 5.

### Transcriptome Sequencing and Analysis

For RNA sampling, RNA was extracted from the following greenhouse grown samples (three biological replicates): aerial stem, subterranean stem, aerial chasmogamous flowers (A_CH_F), aerial cleistogamous flowers (A_CL_F), and subterranean cleistogamous flowers (S_CL_F), A_CH_F_seed, A_CL_F_seed, and S_CL_F_seed, which were collected from independent plants to construct RNA sequencing libraries. All of these fresh tissues were harvested, immediately frozen in liquid nitrogen, and then stored at −80°C until extraction.

All the libraries were sequenced using the Illumina HiSeq X-ten platform with PE150. The paired-end reads that were retained after quality control were mapped to the genome by using HISAT2 (v.2.0.4; Kim et al., 2015) with default parameters. Fragments per kilobase of exon per million mapped reads (FPKM) was used to represent the expression level of each gene. Differentially expressed gene (DEG) was detected using DESeq2 package (Anders and Huber, 2010) in R software, and DEGs with an adjusted value of *p*<0.05 were considered significant. To perform a weighted correlation network analysis (WGCNA) of gene co-expression, we used the WGCNA package (Langfelder and Horvath, 2008) in R software with the significant DEGs.

### Identification of MADS-Box TFs

The MADS-box gene family belongs to the plant TFs. Firstly, we used the ITAK (Zheng et al., 2016) program to identify all TFs of *A. edgeworthii*. Secondly, we selected the MADS-box gene family from the identified TFs. For the evolutionary analysis of type II subfamily of MADS-box, we aligned the genes from *A. edgeworthii*, *A. thaliana*, *G. max*, and *G. soja* by using MAFFT (Katoh and Standley, 2013). We used FastTree software (v.2.7.6; Price et al., 2010) to construct a phylogenetic tree of MADS-box genes and Evolview software (Subramanian et al., 2019) for editing (Supplementary Figure 9).

### Paraffin Section and SEM

We observed the structure of seeds by using the paraffin section method. The plant samples were soaked in the FAA-fixed liquid and the fixed samples were dehydrated and stained using saffron solid green dye. Finally, the samples were embedded in parafilm, sliced, and observed under a light microscope (Ellison et al., 2016). For scanning electron microscopy (SEM) observations, dried seeds were mounted on aluminum stubs and coated with gold sputter. Subsequently, the examination was performed using the Hitachi S3400N SEM (Japan; Arabi et al., 2017).

## Results

### Genome Assembly and Annotation

*Amphicarpaea edgeworthii* has a diploid genome (2n=2x=22; Supplementary Figure 1). Based on 17-mer analysis we estimate the genome size of *A. edgeworthii* to be 360.91Mb (Supplementary Table 1 and Figure 2). Thereafter, we sequenced the genome of *A. edgeworthii* by using a combination of PacBio, Illumina, and 10× Genomics libraries that resulted in the generation of a 343.78-Mbp genome (contig N50 length=1.44Mb, scaffold N50 length=2.4Mb; Table 1 and Supplementary Tables 2, 3, and 4). Finally, we assembled a chromosome-level genome by using Hi-C technology. We used a total of 5.27 million reads from Hi-C libraries and mapped approximately 90.61% of the assembled sequences to 11 pseudochromosomes, with the longest scaffold length of 32.05Mb (Table 1, Figure 2 and Supplementary Figure 3). Results indicated that the *A. edgeworthii* genome was adequately covered by the assembly. We evaluated the completeness of the genome assembly by mapping the Illumina paired-end reads to our assembly utilizing Burrows–Wheeler Alignor (BWA; Li and Durbin, 2009), with 98.70% of mapping rate and 94.04% of coverage (Supplementary Table 5). Then, we used both the Core Eukaryotic Gene Mapping Approach (CEGMA; Parra et al., 2007) and Benchmarking Universal Single-Copy Orthologs (BUSCO; Simão et al., 2015) to assess the integrity of the assembly. In the CEGMA assessment, 238 (95.97%) of 248 core eukaryotic genes were assembled (Supplementary Table 6). Furthermore, 93.4% complete single-copy BUSCOs were detected, which indicated that the assembly was complete (Supplementary Table 7). Overall, the results indicate that the generated assembly was of high quality.

**Table 1:** Statistics of the *A. edgeworthii* genome assembly.

Total assembly size (Mb)	343.78
Total number of contigs	1,475
Total number of scaffolds	1,082
Contig N50 length (Mb)	1.44
Maximum contig length (Mb)	7.65
Maximum scaffold length (Mb)	32.05
Scaffold N50 length (Mb)	28.47
Scaffold N90 length (Mb)	23.07
GC content (%)	32.04
Gene number	28,372
Repeat content (%)	51.28

Repeat sequences comprise 51.28% of the assembled genome, with transposable elements (TEs) being the major component (Supplementary Table 8). Among TEs, long terminal repeats (LTRs) were the major component (29.32%; Supplementary Table 9). We combined *de novo* prediction, homology search, and mRNA-seq assisted prediction to predict genes in the *A. edgeworthii* genome, and we obtained 28,372 protein-coding genes (97.2% of which were annotated; Supplementary Tables 10 and 11). Additionally, we identified 2,260 non-coding RNAs, including 471 miRNAs, 701 transfer RNAs, 266 ribosomal RNAs, and 822 small nuclear RNAs (Supplementary Table 12).

### Comparative Genomic and Phylogenomic Analyses

**Figure 3:**
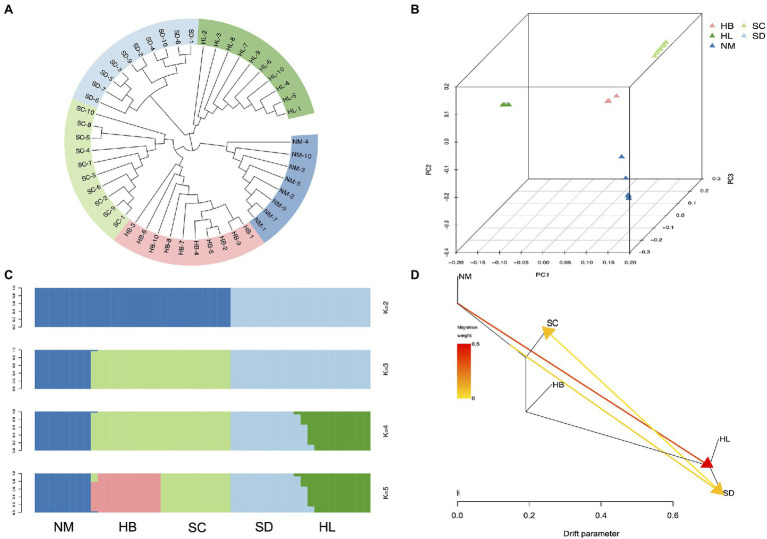
Comparative genomic analysis of *A. edgeworthii* and 11 other plant species. **(A)** Comparison of the number of gene families identified using OrthoMCL. Venn diagram showing unique and shared gene families between the genomes of *A. edgeworthii* and 4 other legume species. **(B)** Expansion and contraction of gene families among 12 plant species. The number of expanded (green) and contracted (red) gene families is shown above the branches. **(C)** The abscissa represents the transversion substitutions at 4-fold degenerate sites (4DTv) distance, and the ordinate indicates the percentage of gene pairs corresponding to the 4DTv values. **(D)** Syntenic analysis. Synteny blocks shared between *A. edgeworthii* and its close relatives *Glycine max* and *Glycine soja*. We identified 383 syntenic blocks between *A. edgeworthii* and *G. max* genomes and 376 synteny blocks between the *A. edgeworthii* and *G. soja* genomes. Gray ribbons connect the matching gene pairs. Colored lines show an example of syntenic blocks found in other species, corresponding to one copy in the *A. edgeworthii* genome and 2 copies in *G. max* and *G. soja* genomes.

To perform similarity-based clustering of homologs, we used the genes of *A. edgeworthii* and 11 other plants with fully-sequenced genomes. The genes of *A. edgeworthii* are shared with other plants and distributed across 13,077 gene families, of which 554 gene families appear to be unique to *A. edgeworthii* (Figure 3A and Supplementary Figure 4). The gene families unique to *A. edgeworthii* are enriched in diverse biosynthesis-related pathways (e.g., phenylpropanoid, isoquinoline alkaloid, flavonoid and isoflavonoid, and ubiquinone and other terpenoid-quinone) and diverse energy metabolism-related pathways (such as the metabolism of carbon compounds, namely starch, sucrose, and galactose; Supplementary Tables 13 and 14), which play crucial roles in plant growth, development, and resistance.

We constructed a phylogenetic tree, inferred from 653 single-copy orthologous genes were extracted from 12 species, and aligned using MUSCLE (Edgar, 2004). Among the sequenced species, soybean (*G. max*) and wild soybean (*G. soja*) were most closely and phylogenetically related to *A. edgeworthii* and were grouped into a single branch, with an estimated divergence time of approximately 14.2 million years ago (Mya; Figure 3B and Supplementary Figure 5). Other branching orders of the tree were consistent with the previously proposed phylogenetic ordering (Lavin et al., 2005).

In addition, we used the CAFE (Computational Analysis of gene Family Evolution) tool, a comparative genomics tool, which showed that the *A. edgeworthii* genome has lost many gene families (*n*=1,950), while only expanding a modest 246 gene families (Figure 3B). Compared with the other two soybean genomes that are in the same clade, the number of contracted gene families was highest in the *A. edgeworthii* genome. Results from gene ontology (GO) and Kyoto Encyclopedia of Genes and Genomes (KEGG) annotations indicated that species-specific expanded genes are considerably enriched in functional and biological process categories, such as photosynthesis (GO:0015979), pathogenesis (GO:0009405), photosynthetic electron transport chain (GO:0009767) and hydrolase activity (GO:0016787; Supplementary Table 15 and Supplementary Figure 6A). Furthermore, KEGG pathway analysis of these expanded gene families revealed significant enrichment of genes involved in photosynthesis and biosynthesis of isoquinoline alkaloids, ubiquinones and other terpenoid-quinones, phenylpropanoids, glucosinolates, and carotenoids and diterpenoids (Supplementary Table 16 and Supplementary Figure 6B). These pathways are closely linked to the biosynthesis of antioxidants and hormones. The expansion of these genes indicated their probable roles related to the accumulation of secondary metabolites (such as hormones and antioxidants) and light-regulated plant growth and development. For the contracted gene families were mostly enriched in the pathways of starch and sucrose metabolism, plant pathogen interaction, plant hormone signal transduction et al. (Supplementary Table 18 and Supplementary Figure 7B), and in the GO terms of catalytic activity (GO:0003824), nucleotide binding (GO:0000166), kinase activity (GO:0016301; Supplementary Table 17 and Supplementary Figure 7A).

Whole-genome duplication (WGD) events are common in plants and are the powerful forces that drive plant genome evolution. According to the abundance of 4DTv (transversion substitutions at 4-fold degenerate sites) values, we evaluated the relative timing of WGD or species divergence events. In general, only one significant peak was seen in the *A. edgeworthii* genome (4DTv=approximately 0.27, Figure 3C). The results suggest that *A. edgeworthii* has undergone a single WGD, not specific to *A. edgeworthii*, but rather part of a pan-legume duplication event in legume species, such as *M. truncatula* and soybean (Young et al., 2011). Compared with *A. edgeworthii*, additional WGDs were noted in soybean and wild soybean. We used MCscan to identify genome synteny blocks within *A. edgeworthii* and other related species. Results suggested that the *A. edgeworthii* genome shares highly conserved syntenic blocks with the genomes of *G. max* and *G. soja*, which were used as the reference genomes. Moreover, 1 syntenic block of *A. edgeworthii* corresponded to 2 syntenic blocks of soybean and wild soybean (Figure 3D).

### Population Structure and Diversification of *A. edgeworthii*

*Amphicarpaea edgeworthii* species are distributed widely in various provinces of China from Heilongjiang and Nei Mongol to Sichuan, with populations on the mountain slopes, roadsides, and fields from full sun to dappled shade between 300m and 3,000m (Alexander et al., 2009). We collected and sequenced 48 individuals from 5 different sites, covering the primary habitats of the entire species distribution (Supplementary Table 19). From individuals of each population, we yielded an average 10.55-fold depth and 89% coverage (Supplementary Tables 20 and 21). After rigorous variant calling and filtering of SNPs, we identified a total of 1,565,692 high-quality SNPs, with a coverage depth of ≥3, Reconfigurable Machine System (RMS) mapping quality of ≥20, minor allele frequency of ≥0.05, and missing frequency of ≤0.1, which were used for subsequent population-based analyses (Supplementary Table 22).

**Figure 4:**
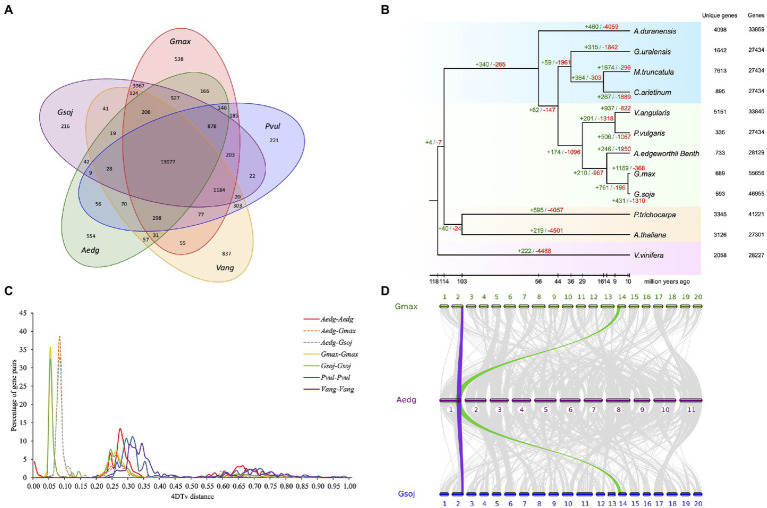
Population structure analysis. **(A)** Neighbor-joining phylogenetic tree of the 48 sequenced individuals. **(B)** Each point refers to an individual, where colors distinguish populations. **(C)** Population genetic structure of all SNPs estimated on the basis of ADMIXTURE analysis with *K*=2 to 5. **(D)** Maximum-likelihood tree and migration events among 5 populations of *A. edgeworthii*. Migration events are colored by their weights.

To elucidate phylogenetic relationships from a genome-wide perspective, an individual-based neighbor-joining (NJ) tree was constructed using TreeBest software based on the p-distance, which resulted in the generation of divergent clades of 5 different populations (Figure 4A). Although HL and SD were phylogenetically closer, SC, HB, and NM also exhibited high proximity. The PCA also recovered these groupings (Figure 4B), and the results corresponded to the NJ tree, in which HL was located near SD. Genetic structure for 48 sequenced individuals were inferred using ADMIXTURE analysis (Alexander et al., 2009), with *K*=2 to 8. Each individual was represented by a stacked column, which was partitioned into 2–8 colored segments, with the length of each segment representing the proportion of the individuals’ genome from *K*=2 to *K*=8 ancestral populations. With *K*=3, the populations of HL and SD were clustered together in a group and SC and HB were clustered together in another group, which suggest that they were extremely closely related. However, the population structure analysis revealed that 5 population clusters (*K*=5) represent an optimal model (Figure 4C and Supplementary Figure 8), which clearly separates the species in different regions. Five distinct clusters (*K*=5) reflected geographic divergence and limited gene flow between certain populations (Figure 4D).

### Gene Co-expression Modules and Clusters Related to Flower Development

**Figure 5:**
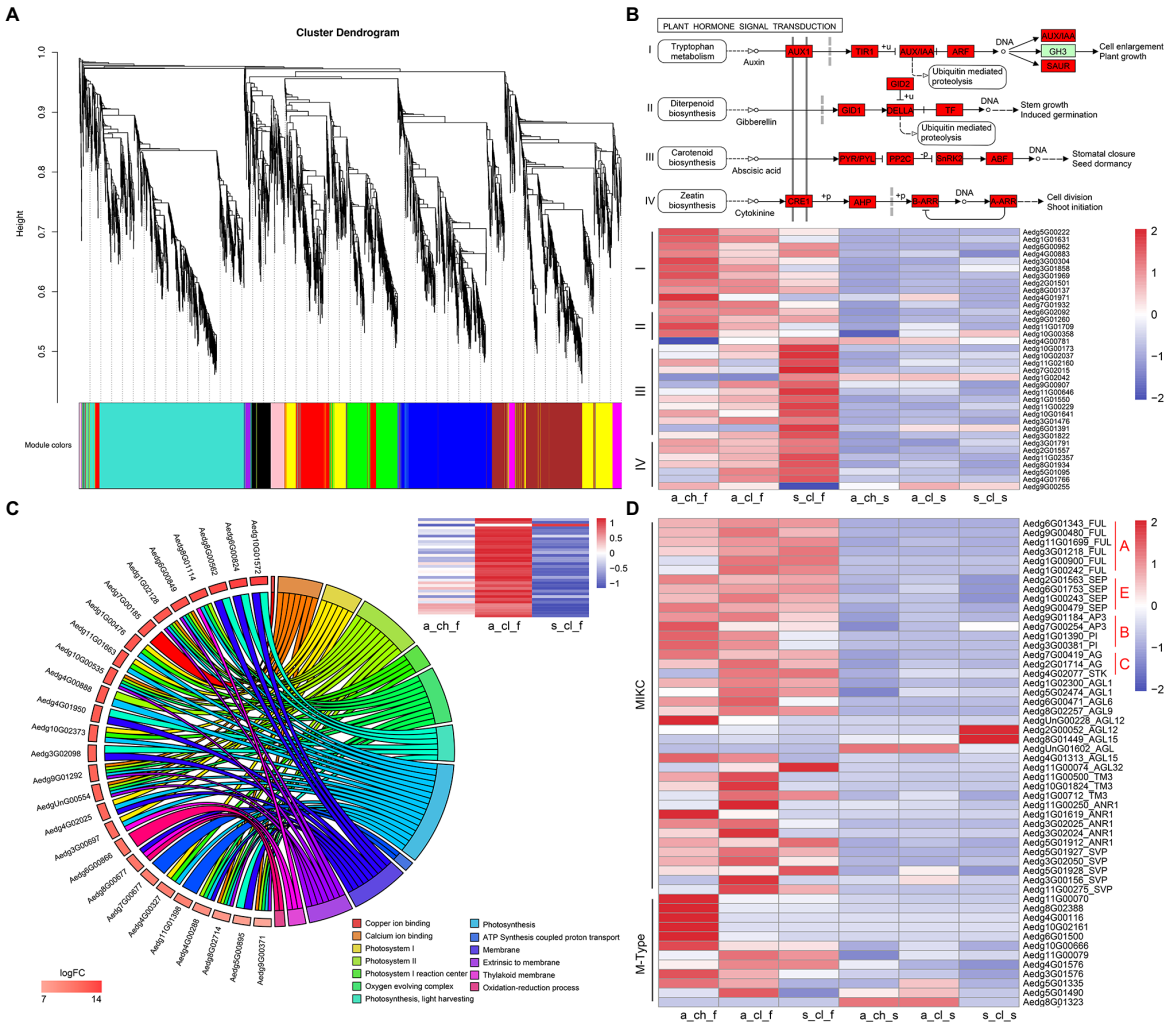
Gene co-expression modules and clusters related to flower development. **(A)** Hierarchical cluster tree showing co-expression modules identified using WGCNA. **(B)** Hormone-enriched expression in MEyellow and MEturquoise. Red boxes indicate the genes that were identified. The heatmap shows the relative FPKM of genes from the module. **(C)** The chord plot of GO terms revealing enrichment to photosynthesis pathway genes. The heatmap shows the relative FPKM of genes from photosynthesis in the 3 types of flower. **(D)** Expression profiling of MADS-box from various organs of *A. edgeworthii*. Two genes clusters were divided according to the type of MADS-box genes. All expression values were scaled by log_2_ (FPKM+1), where FPKM denotes fragments per kilobase of exon per million mapped reads.

To investigate differences between the 3 types of flowers and their seeds, we performed the comparative transcriptome analysis was performed (Figures 1A–E). We performed the WGCNA of transcript expression in 8 samples, which included 3 types of flowers, 3 types of seeds, and 2 types of stems. A total of 5,343 DEGs identified by comparing different tissue samples were filtered and grouped by topological overlap, which was followed by the generation of gene modules from a dynamic tree cutting. Lastly, 10 gene modules (marked with different colors) were identified by merging modules with similar expression patterns. Of the 10 co-expression modules, 4 modules (MEyellow, MEturquoise, MEblue, and MEbrown) were associated with different types of flower samples (Figure 5A and Supplementary Table 23). Abundance of MEyellow and MEturquoise transcripts correlated with hormone signal transduction pathways in flowers (Figure 5B). Auxin pathway-, gibberellic acid (GA) pathway-, abscisic acid (ABA) pathway-, and cytokinin (CK) pathway-related genes were also identified. These pathways have been shown to play pivotal roles in the regulation of flowering in many plants (Wang et al., 2016; Campos-Rivero et al., 2017; Israeli et al., 2020). Expression analysis of 36 hormone signal transduction pathway genes by RNA sequencing revealed that auxin pathway- and GA pathway-related genes display a similar expression pattern and are highly expressed in A_CH_F, whereas ABA pathway- and CK pathway-related genes are highly expressed in S_CL_F (Figure 5B). Among higher plants, photosynthetic organisms display the adaptation mechanism to a variety of light conditions, which is one of the most important functions, and the light-harvesting chlorophyll a/b-binding protein (Lhc) superfamily plays diverse roles in multiple processes associated with plant growth, development, and abiotic stress response (Chang et al., 2020). In the MEblue, several known photosynthesis genes, namely *FNR* (ferredoxin–NADP+ reductase), *Lcha*, and *Lchb* were detected, which covered almost all or most of the photosynthetic genes (Figure 5C). Because MEblue was enriched in A_CL_F and S_CL_F, we further analyzed the expression and enrichment of the DEGs through the GO and KEGG analysis. The GO enrichment analysis revealed that 27 DEGs are enriched in 13 most significant GO terms, namely photosynthesis process, light-harvesting process, oxidation–reduction process, and ATP synthesis-coupled proton transport process. Interestingly, the 36 DEGs annotated to photosynthesis were significantly upregulated in A_CL_F, as shown in the heatmap (Figure 5C). Additionally, the enrichment of MEbrown in DEGs was mostly related to energy metabolism pathways, for example, oxidative phosphorylation, citrate cycle, carbon metabolism, and pyruvate metabolism. Based on our findings that indicated a significant upregulation of DEGs in A_CH_F (*p*<0.05), we speculate that cleistogamous flowers consume lesser energy than chasmogamous flowers. The requirement of a large amount of carbohydrates during flowering has been documented in previous studies (Kozłowska et al., 2007; Huang et al., 2020).

The MADS-box family has been reported to be a highly conserved gene family involved in the flowering process (Martínez-Castilla and Alvarez-Buylla, 2003; Krizek and Fletcher, 2005; Rijpkema et al., 2007; Schilling et al., 2018). In addition to the critical role of MADS-box genes in flower development, these genes have been considered important for the regulation of root development, seed pigmentation, embryo development, and other processes (Nesi, 2002; Smaczniak et al., 2012). We identified a total of 53 MADS-box genes, of which 13 were type I (M-type) genes and 40 were type II (MIKC-type) genes (Figure 5D and Supplementary Figure 9). Of the 13 M-type genes, one gene was not expressed in any of the tissues, and therefore, it was not represented in the heatmap. All homologs of the ABCE model prototype genes, which include AP1/FUL and AGL6 (A-function for sepal and petal), AP3 and PI (B-function for petal and stamen), AG (C-function for stamen and carpel), and SEP (E-function for interacting with ABC function proteins) have been identified (Rijpkema et al., 2007; Zhang et al., 2020b). Zhang et al. (2006) examined floral ontogeny of *A. edgeworthii* by using SEM and found that the 3 flower morphs do not differ significantly in terms of organ initiation and that only aerial and subterranean flowers diverge at the mid to late development stage. No significant differences were observed in the floral development and morphology between S_CL_F and A_CL_F; however, both S_CL_F and A_CL_F exhibited partial petal and stamen suppression compared with A_CH_F. Because of the unique characteristics of cleistogamous flowers, which are particularly small, sequencing of flowers according to their structures was challenging. As expected, the expression of B-function homologs was downregulated in A_CL_F and S_CL_F compared with that in A_CH_F (Figure 5D). Of 5 M-type genes that are highly expressed in A_CH_F, 4 genes have been identified as paralogous *AGL62*-like gene; however, transgenic evidence is required to confirm their function in future.

### Seed Micromorphology and Its Transcriptome Profiling

**Figure 6:**
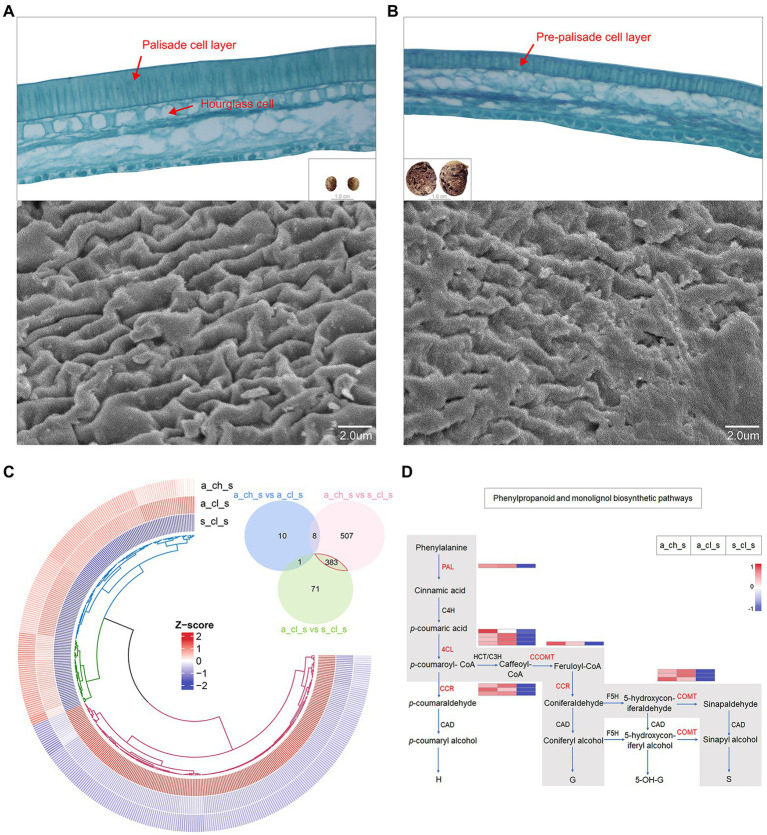
Seed micromorphology and its transcriptome profiling. **(A)** Paraffin section images of an aerial seed and scanning electron microscopy (SEM) details of the testa surface. **(B)** Paraffin section images of a subterranean seed and SEM details of the testa surface. **(C)** Venn plot and circular heatmap between aerial (A_CH_S and A_CL_S) and subterranean seeds (S_CL_S). **(D)** Biosynthetic pathways of phenylpropanoid and monolignol. The gray box indicates the phenylpropanoid and monolignol biosynthetic pathways that are generally accepted for angiosperms with indication of the expression levels of the 5 key genes (in red) in this study by heatmap.

An extreme form of seed heteromorphism is termed amphicarpy (Sadeh et al., 2009; Baskin and Baskin, 2014; Zhang et al., 2017), *A. edgeworthii* bears aerial and subterranean fruits (seeds), which differ in size, mass, as well as in their water permeability and dormancy (Figure 1D), specifically, the seed coat acts as a barrier to water permeability (Willis et al., 2014). To test whether two seed types are structurally different, we used paraffin sections and SEM. We observed a thick and dense palisade cell layer of the seed coat in the aerial seed and clearly visualized the hourglass cell layer. SEM examination revealed that the surface cells are ridged, characterized by periclinal extended projections (Figure 6A). Relative to the aerial seeds, the seed coat of the subterranean seeds consisted of an immature, thin, and loose pre-palisade cell layer, with the seed surface being crumpled irregularly and having a rugose appearance to the surface with no significant ridges; owing to these features the subterranean seeds exhibit more water permeability than aerial seeds (Figure 6B).

We identified DEGs in the 3 types of seeds. A Venn diagram revealed more alternated genes between aerial and subterranean seeds than that between two aerial seeds (Figure 6C), which was also consistent with the phenotype. The analysis of 383 DEGs demonstrated that genes involved in stress response and defense response were significantly upregulated in aerial seeds. For instance, the defensin-like proteins, namely PDF2.1, MOS2, and RPS2, may promote stress tolerance in aerial seeds (Supplementary Table 24). Interestingly, the DEGs annotated to the TCA cycle, oxidation–reduction, starch and sucrose metabolism, and lipid metabolic processes were significantly upregulated in the subterranean seeds (Supplementary Table 25). This further suggested that the size of the subterranean seeds may be associated with these energy metabolism pathways.

The cell wall, mainly composed of lignin and cellulose, is a critical factor that influence the hardness of seeds (Cosgrove, 2005). Monolignols are the principal building blocks of lignin polymer and are synthesized from phenylalanine through the general phenylpropanoid and monolignol-specific pathways (Figure 6D; Boerjan et al., 2003; Fraser and Chapple, 2011; Van Acker et al., 2013). Aerial seeds exhibited a relatively higher transcript accumulation of *PAL*, *4CL*, *CCR*, *CCOMT*, and *COMT* genes, as well as the cellulose synthase, mannan and sucrose synthase enzymes (Supplementary Figure 10). These results indicated that the phenotype of aerial seeds may be correlated with the cellulose as well as the lignin content of the cell wall. Alterations in gene expression patterns may not only enhance a short-term response of organisms under diverse environmental stress conditions but also trigger the long-term adaptation to evolution through enhanced phenotypic variability and robustness (López-Maury et al., 2008). Spread of aerial seeds over long distances has established their population in diverse regions, and their ability to adapt to uncertain external environmental conditions and germinate under right conditions lead to the evolution of new phenotypes (Zhang et al., 2020a). Taken together, these results suggest that molecular adaptation and transcriptional regulation of genes involved in morphogenetic building of aerial seeds may play a major role in their successful survival in a complex external environment.

## Discussion

In this study, we constructed a high-quality chromosome-level genome assembly for *A. edgeworthii* by combining the long-read sequences from PacBio with highly accurate short reads from Illumina sequencing and by using Hi-C technology for super-scaffolding, as well as transcriptomic studies of the three kinds of flowers and fruits produced. The whole genome assembly of *A. edgeworthii* adds to the growing genomic information for the agriculturally critical family Fabaceae, and provides a starting point for a detailed investigation of the genetic bases for the production of aerial and subterranean flowers and fruits by this and other amphicarpic species, and how variation in the relative abundance of these reproductive structures respond to environmental signals.

The MADS-box gene family has been reported to be a complex family of transcription factors involved in the regulation of various functions (Arora et al., 2007; Gramzow and Theissen, 2010; Wei et al., 2018; Schilling et al., 2020). We hypothesized that this gene family forms a complex gene regulatory network governing the production of three flower types and three fruit (seed) types. Different environmental conditions – especially light availability – appear to drive differences in the abundance of aerial vs. subterranean seeds in *A. edgeworthii*, with more open sites resulting in the greater production of aerial seeds (Zhang et al., 2017) which may be better adapted to such conditions (Sadeh et al., 2009; Baskin and Baskin, 2014; Zhang et al., 2020a). We speculate that the seed yield ratio is driven by a signal cascade initiated by light conditions. However, transgenic evidence is required to confirm specific protein–protein interactions and gene function supporting this hypothesis, which is a priority for our future studies.

The genomic data presented here might provide useful tools for dissecting putative cryptic species in Amphicarpaea. We analyzed the evolution and divergence time of this species (Figure 3B and Supplementary Figure 5), and we found that the evolutionary status of *A. edgeworthii* and soybean (*Glycine*) are on the same clade of the phylogenetic tree, and thus, there are extremely close relatives, compared with that of other legume species. We observed independent WGDs in *A. edgeworthii*, cultivated soybean, and wild soybean, and found no significant gene family expansion and numerous contracted gene families in *Amphicarpaea* compared with *Glycine* (Figure 3B). As such, these data may serve as valuable resources for future genomic studies and molecular breeding of soybean. Furthermore, the genome will facilitate future investigations on the phylogenetic relationships between flowering (seed) plants. In addition, the accessibility of the *A. edgeworthii* genome sequence opens up the exploration of deep phylogenetic questions on angiosperms, determination of genome evolution signatures and genetic basis of interesting traits. This assembly will also contribute to the in-depth fundamental comparative genomic analysis for the clarification of evolution mechanism and resolution of genomic evolution between *A. edgeworthii* and other species within the amphicarpic plant family.

## Data Availability Statement

All the raw sequencing data generated during this study have been deposited at NCBI as a BioProject (BioProject PRJNA663436: https://www.ncbi.nlm.nih.gov/bioproject/PRJNA663436) under accession PRJNA663436. Genome assembly, gene annotation and transcriptome files are available at: https://figshare.com/s/565549fb2611c26c229f and https://figshare.com/s/d5fef744486ee2f7bde0.

## Author Contributions

GC and TS conceived and designed the project and the strategy. TS, YY, JY, and JL collected and cultured the plant material, DNA/RNA preparation, library construction and sequencing. TS and MZ worked on genome assembly and annotation, comparative and population genomic analyses, and transcriptome sequencing and analysis. GC, TS, MZ, HC, YC, YB, and GZ contributed substantially to revisions. All authors commented on the manuscript.

## Funding

This research work was funded by the National Key R&D Project of China (2016YFC0500607).

## Conflict of Interest

The authors declare that the research was conducted in the absence of any commercial or financial relationships that could be construed as a potential conflict of interest.

## Publisher’s Note

All claims expressed in this article are solely those of the authors and do not necessarily represent those of their affiliated organizations, or those of the publisher, the editors and the reviewers. Any product that may be evaluated in this article, or claim that may be made by its manufacturer, is not guaranteed or endorsed by the publisher.

## Footnotes

^1^ http://treesoft.sourceforge.net/treebest.-shtml
