# Supplementary material for: Chromosome-Scale Reference Genome of Amphicarpaea edgeworthii: A New Resource for Amphicarpic Plants Research and Complex Flowering Pattern
Source: Front Plant Sci. 2021 Nov 18;12:770660. doi: 10.3389/fpls.2021.770660 (PMC8637744; doi:10.3389/fpls.2021.770660)
Supplement: Supplementary file 1 [file Data_Sheet_1.docx]

**Supplemental Information**

Title: Chromosome-scale reference genome of *Amphicarpaea edgeworthii*: a new resource for amphicarpic plants research and complex flowering pattern

Tingting Song^1+^, Mengyan Zhou^2+^, Yuying Yuan^1^, Jinqiu Yu ^1^, Hua Cai^1^,

Jiawei Li^1^, Yajun Chen^1^, Yan Bai^2^, Gang Zhou^2^ and Guowen Cui^1^*

1 Northeast Agricultural University, 150030, Harbin, China

2 Novogene Bioinformatics Institute, 100083 Beijing, China

+ These authors contributed equally

* Corresponding author:

Guowen Cui, Northeast Agricultural University, Harbin, No. 600 Changjiang Road, Harbin, 150030, China. Tel: +86-0451-55191248.

Email: cgw603@163.com

**Running title: *Amphicarpaea edgeworthii* genome**


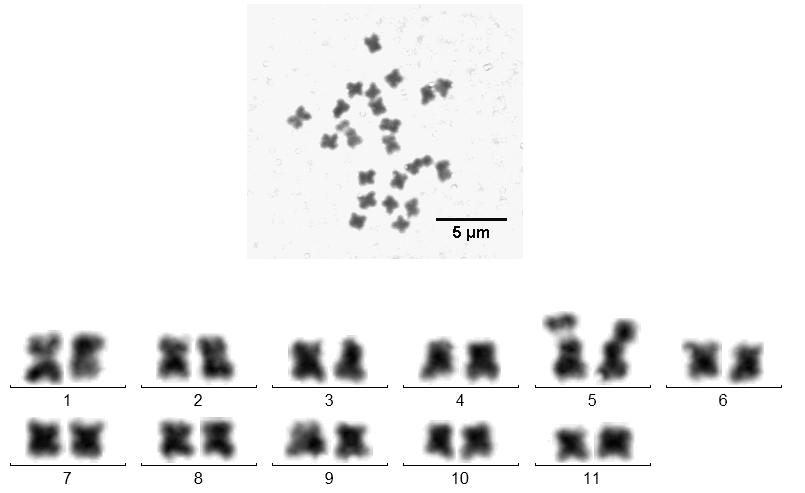


**Supplementary Figure 1. Mitotic metaphase chromosomes of the sequenced individual of *A.edgeworthii*.** The chromosome number is 22, indicating the sequenced individual is diploid. **Related to Figure 2.**

**Supplementary Figure 2.** **17-mer depth distribution of the *A.edgeworthii* genome sequencing reads.** The depth of peak was at 53. **Related to Figure 2.**

**
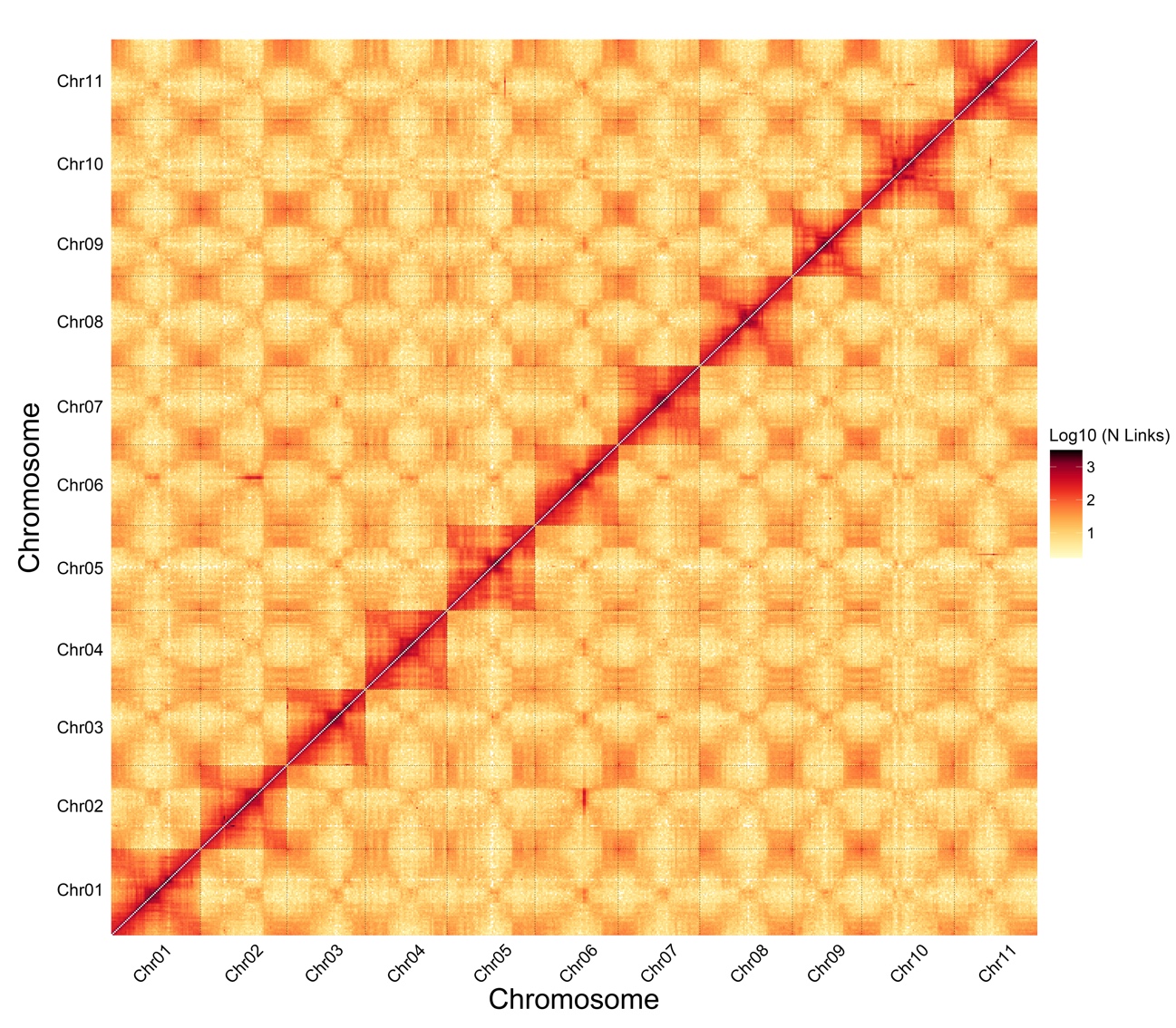
**

**Supplementary Figure 3.** **Hi-C linkage density heat map of assembled contigs.** Showing the contigs were clustered into 11 pseudo-chromosomes (2n=22). **Related to Figure 2.**

**Supplementary Figure 4. Distribution of genes and gene families of 12 plant species we investigated in the study. Related to Figure 3.**

**Supplementary Figure 5. Phylogenetic relationships and divergence times of 12 plant species investigated in the present study.** Numbers in the bracket indicate the 95% confidence interval of the divergence time. **Related to Figure 3.**

**Supplementary Figure 6. A. GO terms enrichment analysis of the top 20 of expanded genes(q-value< 0.05). B. KEGG pathway analysis of the top 10 KEGG enriched of expanded genes(q-value< 0.05).** The color of the point represents the q value, and the size of the point represents the number of enriched genes. **Related to Figure 3.**

**Supplementary Figure 7. A. GO terms enrichment analysis of the top 20 of contracted genes(q-value< 0.05). B. KEGG pathway analysis of the top 10 KEGG enriched of contracted genes(q-value< 0.05).** The color of the point represents the q value, and the size of the point represents the number of enriched genes. **Related to Figure 3.**

**
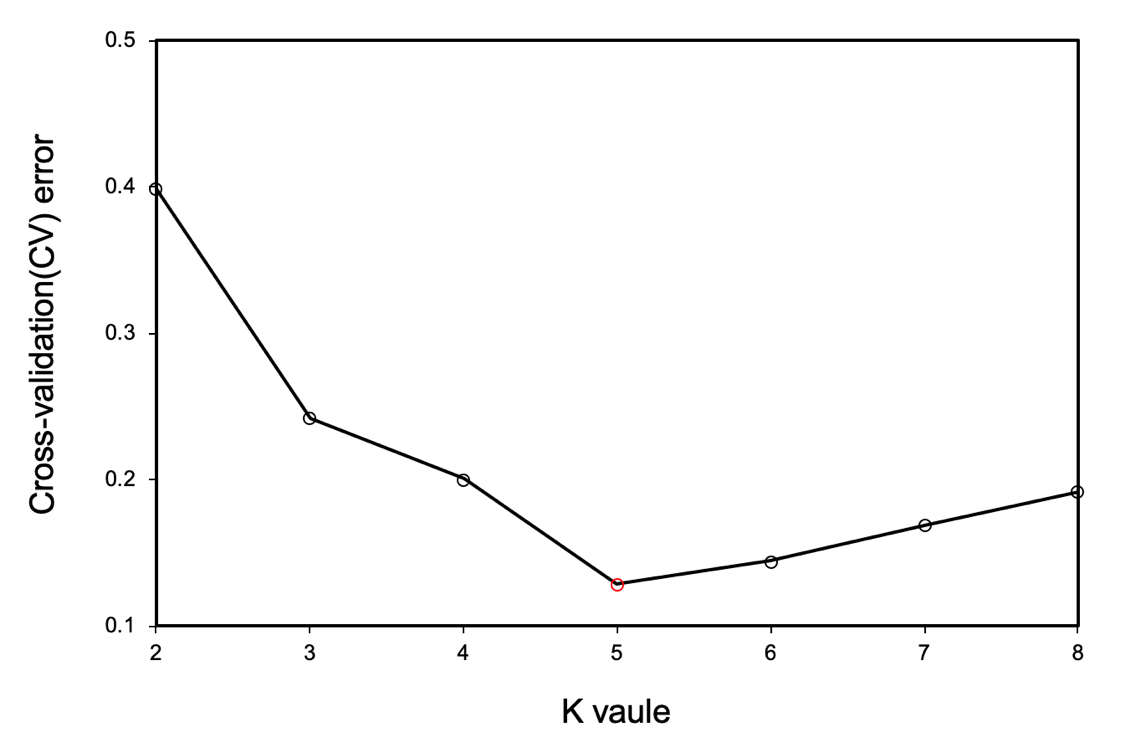
**

**Supplementary Figure 8. The optimal clustering at K = 5 groups were supported based on the CV error. Related to Figure 4.**

**Supplementary Figure 9. Phylogenetic tree constructed using the neighbor-joining method with MADS-box genes from *A.thaliana*, *G. max*, *G. soja*, and *A.edgeworthii*.** Different colors indicate different species; blue is *A.thaliana*, green is *G. max*, yellow is *G. soja* and pink is *A.edgeworthii*. **Related to Figure 5.**

**Supplementary Figure 10. Expression levels of mannan synthase, cellulose synthase and sucrose synthase in different types of seeds. Related to Figure 6.**

**Supplementary Table 1. Statistics for the K-mer analysis. Related to Figure 1.**

| **K-mer** | **K-mer Number** | **K-mer Depth** | **Genome Size (Mb)** | **Heterozygous Ratio (%)** | **Repeat(%)** |
| --- | --- | --- | --- | --- | --- |
| 17 | 19,453,880,408 | 53 | 360.91 | 0.30 | 48.14 |

**Supplementary Table 2. Sequencing data generated from different platforms or strategies. Related to Figure 1.**

| **Platform** | **Insert size** | **Read length (bp)** | **Raw data (G)** | **Sequence coverage (X)** # |
| --- | --- | --- | --- | --- |
|  |  |  |  |  |
| Illumina | 350bp | 150 | 103.96 | 288.05 |
| PacBio | -- | -- | 37.32 | 103.41 |
| 10X Genomics | -- | 150 | 138.63 | 384.11 |
| Hi-C | 350 bp | 150 | 45.38 | 125.76 |
| Total | -- | -- | 325.29 | 901.31 |

# The estimated genome size is ~360.91 Mb, and sequence coverage = Total raw bases/360.91 Mb.

**Supplementary Table 3. Statistics of the assembly of the *A.edgeworthii* genome assembly . Related to Figure 1.**

| **Sample ID** | **Length** | | **Number** | |
| --- | --- | --- | --- | --- |
|  | **Contig (bp)** | **Scaffold (>100 bp)** | **Contig** | **Scaffold (>100 bp)** |
| Total | 343,740,538 | 343,779,838 | 1,475 | 1,082 |
| Max | 7,653,304 | 32,051,318 | - | - |
| Number>=100 | - | - | 1,475 | 1,082 |
| Number>=2000 | - | - | 1,435 | 1,042 |
| N50 | 1,444,032 | 28,474,477 | 67 | 6 |
| N60 | 1,113,772 | 27,686,701 | 93 | 7 |
| N70 | 831,149 | 26,642,412 | 129 | 9 |
| N80 | 448,254 | 26,200,017 | 183 | 10 |
| N90 | 111,078 | 23,069,742 | 315 | 11 |

Note: N50 refers to the length of sequence equal to or greater than the half of total sequences length.

**Supplementary Table 4. Statistics for the GC content. Related to Figure 1.**

|  | **Number** | **% of genome** |
| --- | --- | --- |
| **A** | 116,788,363 | 33.97% |
| **T** | 116,832,048 | 33.98% |
| **C** | 55,078,855 | 16.02% |
| **G** | 55,041,272 | 16.01% |
| **N** | 39,300 | 0.01% |
| **Total** | 343,779,838 | -- |
| **GC** | 110,120,127 | 32.04% |

Note: GC content of the genome without N

**Supplementary Table 5. Statistics of paired-end reads mapping. Related to Figure 1.**

|  | **Sample ID** | **Percentage** |
| --- | --- | --- |
| Reads | Mapping rate (%) | 98.70 |
| Genome | Average sequencing depth | 217.51 |
|  | Coverage (%) | 94.04 |
|  | Coverage at least 4X (%) | 93.06 |
|  | Coverage at least 10X (%) | 92.21 |
|  | Coverage at least 20X (%) | 91.38 |

**Supplementary Table 6. CEGMA results of the *A.edgeworthii* genome. Related to Figure 1.**

| **Species** | **Complete** | | **Complete + partial** | |
| --- | --- | --- | --- | --- |
|  | **Prots** | **% completeness** | **Prots** | **% completeness** |
| *A.edgeworthii* | 235 | 94.76 | 238 | 95.97 |

Note: A protein is classified as complete if the alignment of the predicted protein to the HMM profile represents at least 70% of the original KOG domain, otherwise is classified as partial.

**Supplementary Table 7. BUSCO (Benchmarking Universal Single-Copy Orthologs) results of the *A.edgeworthii* genome. Related to Figure 1.**

| **Species** | **BUSCO notation assessment results** |
| --- | --- |
| *Amphicarpaea edgeworthii* | C: 93.4% [S: 82.9%,D: 10.5%], F: 1.2%, M: 5.4%, n:1440 |

C: Complete BUSCOs

S: Complete and single-copy BUSCOs

D: Complete and duplicated BUSCOs

F: Fragmented BUSCOs

M: Missing BUSCOs

n: Total BUSCO groups searched

**Supplementary Table 8. The prediction of repeats elements in the *A. edgeworthii* genome. Related to Figure 1.**

| **Type** | **Repeat Size (bp)** | **Percent (%)** |
| --- | --- | --- |
| TRF | 12,887,689 | 3.73 |
| Repeat Masker | 136,769,884 | 39.64 |
| Repeat Protein Mask | 37,476,456 | 10.86 |
| Total | 176,927,760 | 51.28 |

**Supplementary Table 9. Categories of TEs predicted in the *A. edgeworthii* genome. Related to Figure 1.**

|  |  | **Repbase + De novo** | | **TE Proteins** | | **Combined TEs** | |
| --- | --- | --- | --- | --- | --- | --- | --- |
|  |  | **Length (bp)** | **% in Genome** | **Length (bp)** | **% in Genome** | **Length (bp)** | **% in Genome** |
| DNA transposon | DNA | 20,929,453 | 6.065557 | 4,642,817 | 1.345533 | 25,572,270 | 7.41109 |
| Retrotransposon | LINE | 17,702,640 | 5.130395 | 8,461,973 | 2.452361 | 26,164,613 | 7.582756 |
|  | SINE | 103,777 | 0.030076 | 0 | 0 | 103,777 | 0.030076 |
|  | LTR | 76,745,004 | 22.241439 | 24,415,265 | 7.075778 | 101,160,269 | 29.317218 |
| Other | Other | 956 | 0.000277 | 0 | 0 | 956 | 0.000277 |
|  | Unknown | 31,698,364 | 9.18649 | 0 | 0 | 31,698,364 | 9.18649 |
|  | Total | 136,769,884 | 39.637226 | 37,476,456 | 10.861037 | 174,246,340 | 50.498263 |

Note: "Other" refer to the repeats that can be classified by Repeat Masker, but not included by the classes above; "Unknown" refer to the repeats that can’t be classified by Repeat Masker.

**Supplementary Table 10. General statistics of predicted protein-coding genes. Related to Figure 1.**

| **Gene set** | | **Number** | **Average transcript length (bp)** | **Average CDS length (bp)** | **Average exon length (bp)** | **Average intron length (bp)** | **Average exons per gene** |
| --- | --- | --- | --- | --- | --- | --- | --- |
| *De novo^#^* | Augustus | 31,232 | 2,707.40 | 1,105.86 | 229.60 | 419.63 | 4.82 |
|  | GlimmerHMM | 48,340 | 5,247.00 | 724.84 | 222.79 | 2,006.82 | 3.25 |
|  | SNAP | 52,948 | 2,402.47 | 752.34 | 173.45 | 494.40 | 4.34 |
|  | Genscan | 37,398 | 4,536.99 | 901.00 | 198.05 | 1,024.43 | 4.55 |
|  | Geneid | 26,795 | 7,340.67 | 1,205.21 | 210.62 | 1,299.24 | 5.72 |
| Homolog^$^ | *Arachis duranensis* | 24,738 | 2,963.43 | 1,266.57 | 261.28 | 441.03 | 4.85 |
|  | *Cicer arietinum* | 26,099 | 2,903.95 | 1,219.39 | 264.54 | 466.71 | 4.61 |
|  | *Phaseolus vulgaris* | 34,065 | 2,361.38 | 1,039.08 | 247.95 | 414.92 | 4.19 |
|  | *Trifolium pratense* | 25,178 | 2,709.65 | 1,172.09 | 260.70 | 439.81 | 4.50 |
|  | *Medicago truncatula* | 35,368 | 2,220.26 | 976.45 | 250.90 | 430.11 | 3.89 |
|  | *Glycine max* | 35,088 | 2,478.73 | 1,037.27 | 248.55 | 454.25 | 4.17 |
| RNA_Seq | Cufflinks | 45,660 | 5,335.79 | 2,257.92 | 327.00 | 521.24 | 6.90 |
|  | PASA | 86,731 | 2,738.81 | 1,022.99 | 202.66 | 423.90 | 5.05 |
| EVM | | 35,144 | 2,699.21 | 1,042.15 | 224.52 | 455.03 | 4.64 |
| PASA update | | 34,789 | 2,708.97 | 1,065.56 | 227.66 | 446.52 | 4.68 |
| Final set + | | 28,372 | 2,968.57 | 1,152.33 | 224.42 | 439.26 | 5.13 |

# Statistics calculated from the gene set predicted form each method.

$ Statistics calculated from the gene set predicted by homolog proteins from each species.

+ Statistics calculated from the *Amphicarpaea edgeworthii* genome.

**Supplementary Table 11. General statistics of the mapping rate to functional database of protein-coding genes. Related to Figure 1.**

| **Database** | | **Annotated Number** | **Annotated Percent (%)** |
| --- | --- | --- | --- |
| NR | | 27,565 | 97.2 |
| Swiss-Prot | | 21,711 | 76.5 |
| KEGG | | 20,507 | 72.3 |
| InterPro | All | 23,127 | 81.5 |
|  | Pfam | 21,558 | 76.0 |
|  | GO | 15,609 | 55.0 |
| Annotated | | 27,586 | 97.2 |
| Total | | 28,372 | - |

**Supplementary Table 12. General statistics of non-coding RNA of the genome. Related to Figure 1.**

| **Type** | | **Number** | **Average length (bp)** | **Total length (bp)** | **% of genome** |
| --- | --- | --- | --- | --- | --- |
| miRNA | | 471 | 114.66 | 54,007 | 0.01565 |
| tRNA | | 701 | 75.22 | 52,730 | 0.01528 |
| rRNA | rRNA | 133 | 196.82 | 26,177 | 0.00759 |
|  | 18S | 16 | 848.25 | 13,572 | 0.00393 |
|  | 28S | 26 | 127.92 | 3,326 | 0.00096 |
|  | 5.8S | 9 | 138.11 | 1,243 | 0.00036 |
|  | 5S | 82 | 98.00 | 8,036 | 0.00233 |
| snRNA | snRNA | 411 | 111.01 | 45,626 | 0.01322 |
|  | CD-box | 277 | 97.29 | 26,949 | 0.00781 |
|  | HACA-box | 48 | 130.71 | 6,274 | 0.00182 |
|  | splicing | 86 | 144.22 | 12,403 | 0.00360 |

**Supplementary Table 13. GO enrichment of unique gene families in *A.edgeworthii.* Related to Figure 2.**

| **GO_ID** | **GO_Term** | **GO_Class** | **Pvalue** | **Adjuste Pvalue** | **Number** |
| --- | --- | --- | --- | --- | --- |
| GO:0004857 | enzyme inhibitor activity | MF | 1.99E-10 | 1.06E-07 | 20 |
| GO:0004866 | endopeptidase inhibitor activity | MF | 2.53E-06 | 0.00010421 | 9 |
| GO:0016149 | translation release factor activity, codon specific | MF | 3.26E-06 | 0.00012475 | 5 |
| GO:0006415 | translational termination | BP | 2.33E-05 | 0.00047988 | 5 |
| GO:0003964 | RNA-directed DNA polymerase activity | MF | 5.62E-05 | 0.00103657 | 6 |
| GO:0006278 | RNA-dependent DNA replication | BP | 5.62E-05 | 0.00103657 | 6 |
| GO:0030599 | pectinesterase activity | MF | 0.00010834 | 0.00184709 | 11 |
| GO:0031418 | L-ascorbic acid binding | MF | 0.00011393 | 0.00184709 | 5 |
| GO:0048544 | recognition of pollen | BP | 0.00015341 | 0.00205188 | 6 |
| GO:0051704 | multi-organism process | BP | 0.00041519 | 0.00411349 | 7 |
| GO:0008135 | translation factor activity, nucleic acid binding | MF | 0.00050349 | 0.00437069 | 7 |
| GO:0006412 | translation | BP | 0.00066888 | 0.00534104 | 20 |
| GO:0048038 | quinone binding | MF | 0.00080154 | 0.00621485 | 4 |
| GO:0030246 | carbohydrate binding | MF | 0.00146724 | 0.01033532 | 10 |
| GO:0008083 | growth factor activity | MF | 0.0014682 | 0.01033532 | 3 |
| GO:0007154 | cell communication | BP | 0.00152877 | 0.01048579 | 18 |
| GO:0003723 | RNA binding | MF | 0.00161301 | 0.01092354 | 19 |
| GO:0005198 | structural molecule activity | MF | 0.00178937 | 0.01181871 | 18 |
| GO:0008283 | cell proliferation | BP | 0.00192628 | 0.01256779 | 3 |
| GO:0005786 | signal recognition particle, endoplasmic reticulum targeting | CC | 0.00273679 | 0.01702538 | 2 |
| GO:0030942 | endoplasmic reticulum signal peptide binding | MF | 0.00273679 | 0.01702538 | 2 |
| GO:0043086 | negative regulation of catalytic activity | BP | 0.00484572 | 0.02728905 | 5 |
| GO:0016651 | oxidoreductase activity, acting on NAD(P)H | MF | 0.00526792 | 0.02935769 | 5 |
| GO:0005200 | structural constituent of cytoskeleton | MF | 0.00549417 | 0.0303029 | 3 |
| GO:0004612 | phosphoenolpyruvate carboxykinase (ATP) activity | MF | 0.00664744 | 0.03441425 | 2 |
| GO:0004450 | isocitrate dehydrogenase (NADP+) activity | MF | 0.00664744 | 0.03441425 | 2 |
| GO:0006102 | isocitrate metabolic process | BP | 0.00664744 | 0.03441425 | 2 |
| GO:0042802 | identical protein binding | MF | 0.00668987 | 0.03441425 | 5 |
| GO:0033926 | glycopeptide alpha-N-acetylgalactosaminidase activity | MF | 0.00917352 | 0.04384501 | 2 |
| GO:0005874 | microtubule | CC | 0.01008006 | 0.04730552 | 3 |
| GO:0050664 | oxidoreductase activity | MF | 0.0152813 | 0.0659314 | 2 |
| GO:0005801 | cis-Golgi network | CC | 0.0152813 | 0.0659314 | 2 |
| GO:0008234 | cysteine-type peptidase activity | MF | 0.01613207 | 0.06904527 | 4 |
| GO:0006094 | gluconeogenesis | BP | 0.01883033 | 0.07932463 | 2 |
| GO:0071822 | protein complex subunit organization | BP | 0.02080795 | 0.08563273 | 7 |
| GO:0004575 | sucrose alpha-glucosidase activity | MF | 0.02170005 | 0.08713613 | 1 |
| GO:0030529 | ribonucleoprotein complex | CC | 0.02182475 | 0.08713613 | 15 |
| GO:0004553 | hydrolase activity, hydrolyzing O-glycosyl compounds | MF | 0.02331935 | 0.0910646 | 16 |
| GO:0055114 | oxidation-reduction process | BP | 0.02602305 | 0.09618706 | 35 |
| GO:0016757 | transferase activity, transferring glycosyl groups | MF | 0.02657003 | 0.09618706 | 16 |
| GO:0008312 | 7S RNA binding | MF | 0.02684105 | 0.09618706 | 2 |
| GO:0008131 | primary amine oxidase activity | MF | 0.02684105 | 0.09618706 | 2 |
| GO:0004650 | polygalacturonase activity | MF | 0.02773244 | 0.09627572 | 4 |
| GO:0006952 | defense response | BP | 0.02792478 | 0.09627572 | 6 |
| GO:0003735 | structural constituent of ribosome | MF | 0.02858102 | 0.09627572 | 13 |
| GO:0005840 | ribosome | CC | 0.02858102 | 0.09627572 | 13 |
| GO:0016984 | ribulose-bisphosphate carboxylase activity | MF | 0.04293057 | 0.12953681 | 1 |
| GO:0005525 | GTP binding | MF | 0.05029749 | 0.14624542 | 10 |
| GO:0006614 | SRP-dependent cotranslational protein targeting to membrane | BP | 0.05152554 | 0.14820518 | 2 |
| GO:0003993 | acid phosphatase activity | MF | 0.06298729 | 0.16788378 | 2 |
| GO:0000055 | ribosomal large subunit export from nucleus | BP | 0.0637017 | 0.16788378 | 1 |
| GO:0042273 | ribosomal large subunit biogenesis | BP | 0.0637017 | 0.16788378 | 1 |
| GO:0008236 | serine-type peptidase activity | MF | 0.06583664 | 0.17181757 | 8 |
| GO:0042545 | cell wall modification | BP | 0.07116153 | 0.18129246 | 4 |
| GO:0016706 | oxidoreductase activity one donor | MF | 0.07188415 | 0.18140576 | 6 |
| GO:0015934 | large ribosomal subunit | CC | 0.07521362 | 0.18803405 | 2 |
| GO:0004252 | serine-type endopeptidase activity | MF | 0.07840751 | 0.19510706 | 5 |
| GO:0016491 | oxidoreductase activity | MF | 0.08505321 | 0.20668657 | 37 |
| GO:0016831 | carboxy-lyase activity | MF | 0.08579153 | 0.20674985 | 3 |
| GO:0004523 | RNA-DNA hybrid ribonuclease activity | MF | 0.08811819 | 0.20767943 | 2 |
| GO:0016760 | cellulose synthase (UDP-forming) activity | MF | 0.08811819 | 0.20767943 | 2 |
| GO:0030145 | manganese ion binding | MF | 0.08811819 | 0.20767943 | 2 |
| GO:0003924 | GTPase activity | MF | 0.09591571 | 0.22118494 | 7 |
| GO:0050896 | response to stimulus | BP | 0.10094088 | 0.2265293 | 26 |
| GO:0015095 | magnesium ion transmembrane transporter activity | MF | 0.10162062 | 0.2265293 | 2 |
| GO:0015693 | magnesium ion transport | BP | 0.10162062 | 0.2265293 | 2 |
| GO:0009507 | chloroplast | CC | 0.10162062 | 0.2265293 | 2 |
| GO:0004521 | endoribonuclease activity | MF | 0.10307244 | 0.22876243 | 3 |
| GO:0009522 | photosystem I | CC | 0.10857246 | 0.23516707 | 2 |
| GO:0004185 | serine-type carboxypeptidase activity | MF | 0.11218914 | 0.23817931 | 3 |
| GO:0008168 | methyltransferase activity | MF | 0.11358497 | 0.24018957 | 10 |
| GO:0007165 | signal transduction | BP | 0.11835795 | 0.24448457 | 12 |
| GO:0000287 | magnesium ion binding | MF | 0.12809679 | 0.25959008 | 4 |
| GO:0030244 | cellulose biosynthetic process | BP | 0.13012532 | 0.26171822 | 2 |
| GO:0005576 | extracellular region | CC | 0.14762528 | 0.29036589 | 4 |
| GO:0051258 | protein polymerization | BP | 0.15255481 | 0.29896272 | 2 |
| GO:0016051 | carbohydrate biosynthetic process | BP | 0.15574201 | 0.3040948 | 4 |
| GO:0070011 | peptidase activity, acting on L-amino acid peptides | MF | 0.15908188 | 0.30836523 | 14 |
| GO:0006413 | translational initiation | BP | 0.1601912 | 0.30873885 | 2 |
| GO:0008107 | galactoside 2-alpha-L-fucosyltransferase activity | MF | 0.16100587 | 0.30873885 | 1 |
| GO:0016758 | transferase activity, transferring hexosyl groups | MF | 0.17129409 | 0.3226843 | 11 |
| GO:0008171 | O-methyltransferase activity | MF | 0.17566264 | 0.32836805 | 2 |
| GO:0033897 | ribonuclease T2 activity | MF | 0.17922144 | 0.32836805 | 1 |
| GO:0047134 | protein-disulfide reductase activity | MF | 0.17922144 | 0.32836805 | 1 |
| GO:0015977 | carbon fixation | BP | 0.17922144 | 0.32836805 | 1 |
| GO:0006694 | steroid biosynthetic process | BP | 0.17922144 | 0.32836805 | 1 |
| GO:0003950 | NAD+ ADP-ribosyltransferase activity | MF | 0.17922144 | 0.32836805 | 1 |
| GO:0045735 | nutrient reservoir activity | MF | 0.18348486 | 0.33213772 | 2 |
| GO:0006913 | nucleocytoplasmic transport | BP | 0.19429692 | 0.34649617 | 3 |
| GO:0009405 | pathogenesis | BP | 0.19704267 | 0.34791363 | 1 |
| GO:0005506 | iron ion binding | MF | 0.19959094 | 0.3512538 | 10 |
| GO:0009308 | amine metabolic process | BP | 0.20722571 | 0.36349428 | 2 |
| GO:0000272 | polysaccharide catabolic process | BP | 0.21447808 | 0.37014766 | 1 |
| GO:0016161 | beta-amylase activity | MF | 0.21447808 | 0.37014766 | 1 |
| GO:0016482 | cytoplasmic transport | BP | 0.21894343 | 0.37543185 | 5 |
| GO:0003743 | translation initiation factor activity | MF | 0.22322612 | 0.3803375 | 2 |
| GO:0016043 | cellular component organization | BP | 0.22650818 | 0.38348695 | 14 |
| GO:0046914 | transition metal ion binding | MF | 0.2399594 | 0.39501009 | 39 |
| GO:0051082 | unfolded protein binding | MF | 0.24738552 | 0.40598544 | 2 |
| GO:0051287 | NAD binding | MF | 0.2554626 | 0.41668442 | 2 |
| GO:0050660 | flavin adenine dinucleotide binding | MF | 0.26303483 | 0.42468773 | 3 |
| GO:0004497 | monooxygenase activity | MF | 0.26354454 | 0.42468773 | 2 |
| GO:0071840 | cellular component organization or biogenesis | BP | 0.2749195 | 0.43905054 | 15 |
| GO:0016705 | oxidoreductase activity | MF | 0.28107881 | 0.44359046 | 11 |
| GO:0004601 | peroxidase activity | MF | 0.28695066 | 0.4515253 | 4 |
| GO:0005618 | cell wall | CC | 0.30285441 | 0.468538 | 4 |
| GO:0006869 | lipid transport | BP | 0.31192569 | 0.4740916 | 2 |
| GO:0007264 | small GTPase mediated signal transduction | BP | 0.31931482 | 0.48122092 | 4 |
| GO:0042546 | cell wall biogenesis | BP | 0.39646831 | 0.58919595 | 1 |
| GO:0071554 | cell wall organization or biogenesis | BP | 0.42054485 | 0.62152346 | 5 |
| GO:0043232 | intracellular non-membrane-bounded organelle | CC | 0.43315984 | 0.6340842 | 16 |
| GO:0030247 | polysaccharide binding | MF | 0.43496991 | 0.6340842 | 1 |
| GO:0005976 | polysaccharide metabolic process | BP | 0.46378615 | 0.67060971 | 3 |
| GO:0006950 | response to stress | BP | 0.47656993 | 0.68355205 | 12 |
| GO:0006511 | ubiquitin-dependent protein catabolic process | BP | 0.4840457 | 0.68873523 | 3 |
| GO:0046872 | metal ion binding | MF | 0.51654927 | 0.72386492 | 53 |
| GO:1902582 | single-organism intracellular transport | BP | 0.51709949 | 0.72386492 | 3 |
| GO:0030036 | actin cytoskeleton organization | BP | 0.54647329 | 0.72522206 | 1 |
| GO:0006184 | GTP catabolic process | BP | 0.54687742 | 0.72522206 | 4 |
| GO:0016788 | hydrolase activity, acting on ester bonds | MF | 0.56937541 | 0.73578706 | 16 |
| GO:0016787 | hydrolase activity | MF | 0.62862706 | 0.80075114 | 60 |
| GO:0005737 | cytoplasm | CC | 0.63095083 | 0.80180212 | 22 |
| GO:0006855 | drug transmembrane transport | BP | 0.65316577 | 0.82029034 | 2 |
| GO:0015238 | drug transmembrane transporter activity | MF | 0.65316577 | 0.82029034 | 2 |
| GO:0033036 | macromolecule localization | BP | 0.67892571 | 0.8388574 | 10 |
| GO:0015979 | photosynthesis | BP | 0.69886404 | 0.85074922 | 2 |
| GO:0044444 | cytoplasmic part | CC | 0.71006139 | 0.85752335 | 17 |
| GO:0008270 | zinc ion binding | MF | 0.72884546 | 0.87428772 | 25 |
| GO:0007017 | microtubule-based process | BP | 0.74300619 | 0.88928034 | 3 |
| GO:0009057 | macromolecule catabolic process | BP | 0.79125533 | 0.91628052 | 4 |
| GO:0035556 | intracellular signal transduction | BP | 0.81284575 | 0.93320274 | 5 |
| GO:0015031 | protein transport | BP | 0.81569966 | 0.93447391 | 8 |
| GO:0005975 | carbohydrate metabolic process | BP | 0.82307762 | 0.94091138 | 17 |

| **Supplementary Table 14. KEGG enrichment of unique gene families in *A.edgeworthii*. Related to Figure 2.** | | | | |
| --- | --- | --- | --- | --- |
| **Map ID** | **MapTitle** | **P-value** | **Adjusted P-value** | **Gene Number** |
| map00460 | Cyanoamino acid metabolism | 5.19499E-09 | 6.10E-07 | 19 |
| map00940 | Phenylpropanoid biosynthesis | 0.000129789 | 0.003388924 | 20 |
| map00950 | Isoquinoline alkaloid biosynthesis | 0.000529429 | 0.009699399 | 10 |
| map00500 | Starch and sucrose metabolism | 0.000891709 | 0.013162755 | 33 |
| map04075 | Plant hormone signal transduction | 0.000896188 | 0.013162755 | 5 |
| map00130 | Ubiquinone and other terpenoid-quinone biosynthesis | 0.001005973 | 0.013906094 | 9 |
| map00944 | Flavone and flavonol biosynthesis | 0.001569891 | 0.019417074 | 5 |
| map00350 | Tyrosine metabolism | 0.002814362 | 0.031494046 | 11 |
| map01210 | 2-Oxocarboxylic acid metabolism | 0.004422138 | 0.043300099 | 11 |
| map00943 | Isoflavonoid biosynthesis | 0.01077959 | 0.083980542 | 6 |
| map02010 | ABC transporters | 0.013467362 | 0.098900941 | 9 |
| map00960 | Tropane, piperidine and pyridine alkaloid biosynthesis | 0.021815649 | 0.150784635 | 6 |
| map00440 | Phosphonate and phosphinate metabolism | 0.026458477 | 0.172715059 | 3 |
| map00941 | Flavonoid biosynthesis | 0.02876484 | 0.17871599 | 8 |
| map00966 | Glucosinolate biosynthesis | 0.033660339 | 0.202825117 | 3 |
| map00603 | Glycosphingolipid biosynthesis - globo and isoglobo series | 0.055592662 | 0.28400599 | 3 |
| map00562 | Inositol phosphate metabolism | 0.094079415 | 0.395369562 | 1 |
| map00270 | Cysteine and methionine metabolism | 0.094397482 | 0.395369562 | 1 |
| map04141 | Protein processing in endoplasmic reticulum | 0.095355733 | 0.395369562 | 19 |
| map00030 | Pentose phosphate pathway | 0.095898149 | 0.395369562 | 1 |
| map00904 | Diterpenoid biosynthesis | 0.113132752 | 0.44119462 | 5 |
| map03010 | Ribosome | 0.118972954 | 0.443788003 | 19 |
| map00561 | Glycerolipid metabolism | 0.121674044 | 0.44677188 | 3 |
| map00330 | Arginine and proline metabolism | 0.136133691 | 0.484718445 | 6 |
| map04712 | Circadian rhythm - plant | 0.141404544 | 0.495971161 | 2 |
| map00220 | Arginine biosynthesis | 0.14486598 | 0.49856028 | 4 |
| map00620 | Pyruvate metabolism | 0.148507318 | 0.49856028 | 8 |
| map00190 | Oxidative phosphorylation | 0.154520551 | 0.51144126 | 2 |
| map03060 | Protein export | 0.174750115 | 0.54755036 | 4 |
| map04146 | Peroxisome | 0.187780389 | 0.561516643 | 2 |
| map00360 | Phenylalanine metabolism | 0.210322041 | 0.595490115 | 5 |
| map03013 | RNA transport | 0.224875359 | 0.629115587 | 7 |
| map04120 | Ubiquitin mediated proteolysis | 0.23322596 | 0.644801182 | 12 |
| map04070 | Phosphatidylinositol signaling system | 0.240536551 | 0.657280111 | 2 |
| map00670 | One carbon pool by folate | 0.263835584 | 0.687015688 | 2 |
| map00630 | Glyoxylate and dicarboxylate metabolism | 0.266009394 | 0.687015688 | 5 |
| map00240 | Pyrimidine metabolism | 0.279323382 | 0.698308454 | 5 |
| map03018 | RNA degradation | 0.294251942 | 0.721236592 | 14 |
| map00942 | Anthocyanin biosynthesis | 0.294632821 | 0.721236592 | 1 |
| map00945 | Stilbenoid, diarylheptanoid and gingerol biosynthesis | 0.309930119 | 0.730626003 | 4 |
| map00780 | Biotin metabolism | 0.310464399 | 0.730626003 | 2 |
| map00020 | Citrate cycle (TCA cycle) | 0.314013729 | 0.730626003 | 4 |
| map00061 | Fatty acid biosynthesis | 0.314013729 | 0.730626003 | 4 |
| map00290 | Valine, leucine and isoleucine biosynthesis | 0.32204518 | 0.734763274 | 2 |
| map00051 | Fructose and mannose metabolism | 0.332416415 | 0.738220336 | 3 |
| map00903 | Limonene and pinene degradation | 0.333576861 | 0.738220336 | 2 |
| map00600 | Sphingolipid metabolism | 0.336125855 | 0.738220336 | 2 |
| map04145 | Phagosome | 0.344112206 | 0.741893289 | 7 |
| map04626 | Plant-pathogen interaction | 0.356988363 | 0.762656958 | 21 |
| map03430 | Mismatch repair | 0.36112362 | 0.762982279 | 4 |
| map00564 | Glycerophospholipid metabolism | 0.365463472 | 0.762982279 | 5 |
| map03410 | Base excision repair | 0.373374307 | 0.762982279 | 1 |
| map00052 | Galactose metabolism | 0.387012556 | 0.773757677 | 9 |
| map03008 | Ribosome biogenesis in eukaryotes | 0.390175538 | 0.773757677 | 8 |
| map00040 | Pentose and glucuronate interconversions | 0.404807385 | 0.78619616 | 11 |
| map00260 | Glycine, serine and threonine metabolism | 0.431243046 | 0.791886337 | 5 |
| map03440 | Homologous recombination | 0.431243046 | 0.791886337 | 5 |
| map03030 | DNA replication | 0.438458164 | 0.791886337 | 5 |
| map00010 | Glycolysis / Gluconeogenesis | 0.443000076 | 0.791886337 | 5 |
| map00230 | Purine metabolism | 0.443688444 | 0.791886337 | 7 |
| map00100 | Steroid biosynthesis | 0.497820039 | 0.853924884 | 3 |
| map00400 | Phenylalanine, tyrosine and tryptophan biosynthesis | 0.50317984 | 0.85686422 | 3 |
| map00073 | Cutin, suberine and wax biosynthesis | 0.523505495 | 0.858295693 | 1 |
| map01212 | Fatty acid metabolism | 0.598324368 | 0.918994944 | 5 |
| map00514 | Other types of O-glycan biosynthesis | 0.614284536 | 0.937382246 | 1 |
| map00970 | Aminoacyl-tRNA biosynthesis | 0.632301762 | 0.951687818 | 3 |
| map00910 | Nitrogen metabolism | 0.658549164 | 0.972583112 | 2 |
| map01230 | Biosynthesis of amino acids | 0.675416071 | 0.979770227 | 16 |
| map00640 | Propanoate metabolism | 0.681771313 | 0.982921831 | 2 |
| map00565 | Ether lipid metabolism | 0.707242498 | 0.992828451 | 2 |
| map04933 | AGE-RAGE signaling pathway in diabetic complications | 0.73231712 | 0.992828451 | 1 |

| **Supplementary Table 15. GO enrichment of expanded gene families in *A.edgeworthii.* Related to Figure 2.**   \| **GO ID** \| **GO Term** \| **GO Class** \| **P-value** \| **Adjusted P-value** \| **Gene Number** \| \| --- \| --- \| --- \| --- \| --- \| --- \| \| GO:0009521 \| photosystem \| CC \| 1.06E-22 \| 4.01E-20 \| 22 \| \| GO:0015979 \| photosynthesis \| BP \| 2.29E-20 \| 1.74E-18 \| 22 \| \| GO:0009522 \| photosystem I \| CC \| 5.60E-15 \| 3.54E-13 \| 12 \| \| GO:0009405 \| pathogenesis \| BP \| 5.68E-11 \| 3.09E-09 \| 7 \| \| GO:0071822 \| protein complex subunit organization \| BP \| 9.00E-11 \| 4.27E-09 \| 17 \| \| GO:0022900 \| electron transport chain \| BP \| 3.51E-10 \| 1.45E-08 \| 9 \| \| GO:0016168 \| chlorophyll binding \| MF \| 1.65E-09 \| 5.06E-08 \| 5 \| \| GO:0000786 \| nucleosome \| CC \| 1.73E-09 \| 5.06E-08 \| 10 \| \| GO:0034622 \| cellular macromolecular complex assembly \| BP \| 2.04E-09 \| 5.55E-08 \| 14 \| \| GO:0006461 \| protein complex assembly \| BP \| 2.67E-09 \| 6.35E-08 \| 14 \| \| GO:0006334 \| nucleosome assembly \| BP \| 5.82E-09 \| 1.11E-07 \| 10 \| \| GO:0009767 \| photosynthetic electron transport chain \| BP \| 9.79E-09 \| 1.49E-07 \| 5 \| \| GO:0043234 \| protein complex \| CC \| 2.59E-08 \| 3.39E-07 \| 35 \| \| GO:0003964 \| RNA-directed DNA polymerase activity \| MF \| 6.21E-08 \| 7.62E-07 \| 8 \| \| GO:0006278 \| RNA-dependent DNA replication \| BP \| 6.21E-08 \| 7.62E-07 \| 8 \| \| GO:0004523 \| RNA-DNA hybrid ribonuclease activity \| MF \| 9.54E-08 \| 1.13E-06 \| 7 \| \| GO:0016788 \| hydrolase activity, acting on ester bonds \| MF \| 2.72E-07 \| 2.95E-06 \| 28 \| \| GO:0006259 \| DNA metabolic process \| BP \| 4.37E-07 \| 4.59E-06 \| 18 \| \| GO:0045735 \| nutrient reservoir activity \| MF \| 2.67E-06 \| 2.53E-05 \| 7 \| \| GO:0042301 \| phosphate ion binding \| MF \| 5.44E-06 \| 4.40E-05 \| 3 \| \| GO:0050821 \| protein stabilization \| BP \| 5.44E-06 \| 4.40E-05 \| 3 \| \| GO:0032991 \| macromolecular complex \| CC \| 7.42E-06 \| 5.64E-05 \| 41 \| \| GO:0004518 \| nuclease activity \| MF \| 1.53E-05 \| 0.000109813 \| 12 \| \| GO:0031012 \| extracellular matrix \| CC \| 1.57E-05 \| 0.000110508 \| 5 \| \| GO:0016043 \| cellular component organization \| BP \| 2.97E-05 \| 0.0001983 \| 20 \| \| GO:0008408 \| 3'-5' exonuclease activity \| MF \| 5.34E-05 \| 0.000322074 \| 5 \| \| GO:0009607 \| response to biotic stimulus \| BP \| 7.67E-05 \| 0.000448564 \| 6 \| \| GO:0022904 \| respiratory electron transport chain \| BP \| 0.000146 \| 0.000817818 \| 4 \| \| GO:0003723 \| RNA binding \| MF \| 0.000173 \| 0.000927545 \| 18 \| \| GO:0019825 \| oxygen binding \| MF \| 0.000181 \| 0.00095412 \| 3 \| \| GO:0008535 \| respiratory chain complex IV assembly \| BP \| 0.000311 \| 0.001494018 \| 2 \| \| GO:0009523 \| photosystem II \| CC \| 0.000348 \| 0.001632591 \| 5 \| \| GO:0004857 \| enzyme inhibitor activity \| MF \| 0.00048 \| 0.002170564 \| 10 \| \| GO:0004806 \| triglyceride lipase activity \| MF \| 0.00059 \| 0.002605776 \| 6 \| \| GO:0008083 \| growth factor activity \| MF \| 0.000809 \| 0.003414357 \| 3 \| \| GO:0052689 \| carboxylic ester hydrolase activity \| MF \| 0.000907 \| 0.00378908 \| 11 \| \| GO:0016149 \| translation release factor activity, codon specific \| MF \| 0.001064 \| 0.004292247 \| 3 \| \| GO:0008283 \| cell proliferation \| BP \| 0.001064 \| 0.004292247 \| 3 \| \| GO:0005488 \| binding \| MF \| 0.002204 \| 0.008290813 \| 182 \| \| GO:0004222 \| metalloendopeptidase activity \| MF \| 0.0028 \| 0.010037641 \| 5 \| \| GO:0004866 \| endopeptidase inhibitor activity \| MF \| 0.0028 \| 0.010037641 \| 5 \| \| GO:0006415 \| translational termination \| BP \| 0.003081 \| 0.01064465 \| 3 \| \| GO:0005200 \| structural constituent of cytoskeleton \| MF \| 0.003081 \| 0.01064465 \| 3 \| \| GO:0016787 \| hydrolase activity \| MF \| 0.003722 \| 0.012627394 \| 64 \| \| GO:0006506 \| GPI anchor biosynthetic process \| BP \| 0.004278 \| 0.014260124 \| 3 \| \| GO:0003676 \| nucleic acid binding \| MF \| 0.0046 \| 0.015200609 \| 52 \| \| GO:0005874 \| microtubule \| CC \| 0.005721 \| 0.018097781 \| 3 \| \| GO:0043623 \| cellular protein complex assembly \| BP \| 0.005763 \| 0.018097781 \| 4 \| \| GO:0043232 \| intracellular non-membrane-bounded organelle \| CC \| 0.009768 \| 0.028335158 \| 19 \| \| GO:0006952 \| defense response \| BP \| 0.011252 \| 0.031210125 \| 6 \| \| GO:0017111 \| nucleoside-triphosphatase activity \| MF \| 0.011922 \| 0.032827662 \| 24 \| \| GO:0030976 \| thiamine pyrophosphate binding \| MF \| 0.012729 \| 0.034303785 \| 2 \| \| GO:0016651 \| oxidoreductase activity, acting on NAD(P)H \| MF \| 0.013253 \| 0.035218832 \| 4 \| \| GO:0008137 \| NADH dehydrogenase (ubiquinone) activity \| MF \| 0.015377 \| 0.03895599 \| 2 \| \| GO:0016846 \| carbon-sulfur lyase activity \| MF \| 0.015377 \| 0.03895599 \| 2 \| \| GO:0008270 \| zinc ion binding \| MF \| 0.017066 \| 0.042110764 \| 29 \| \| GO:0042773 \| ATP synthesis coupled electron transport \| BP \| 0.01824 \| 0.04414777 \| 2 \| \| GO:0000287 \| magnesium ion binding \| MF \| 0.020647 \| 0.049344984 \| 5 \| \| GO:1901363 \| heterocyclic compound binding \| MF \| 0.025618 \| 0.059722124 \| 95 \| \| GO:0097159 \| organic cyclic compound binding \| MF \| 0.025786 \| 0.059747445 \| 95 \| \| GO:0055114 \| oxidation-reduction process \| BP \| 0.035256 \| 0.07880737 \| 29 \| \| GO:0005789 \| endoplasmic reticulum membrane \| CC \| 0.044849 \| 0.094157123 \| 3 \| \| GO:0048038 \| quinone binding \| MF \| 0.047885 \| 0.098893036 \| 2 \| |
| --- | --- | --- | --- | --- | --- | --- | --- | --- | --- | --- | --- | --- | --- | --- | --- | --- | --- | --- | --- | --- | --- | --- | --- | --- | --- | --- | --- | --- | --- | --- | --- | --- | --- | --- | --- | --- | --- | --- | --- | --- | --- | --- | --- | --- | --- | --- | --- | --- | --- | --- | --- | --- | --- | --- | --- | --- | --- | --- | --- | --- | --- | --- | --- | --- | --- | --- | --- | --- | --- | --- | --- | --- | --- | --- | --- | --- | --- | --- | --- | --- | --- | --- | --- | --- | --- | --- | --- | --- | --- | --- | --- | --- | --- | --- | --- | --- | --- | --- | --- | --- | --- | --- | --- | --- | --- | --- | --- | --- | --- | --- | --- | --- | --- | --- | --- | --- | --- | --- | --- | --- | --- | --- | --- | --- | --- | --- | --- | --- | --- | --- | --- | --- | --- | --- | --- | --- | --- | --- | --- | --- | --- | --- | --- | --- | --- | --- | --- | --- | --- | --- | --- | --- | --- | --- | --- | --- | --- | --- | --- | --- | --- | --- | --- | --- | --- | --- | --- | --- | --- | --- | --- | --- | --- | --- | --- | --- | --- | --- | --- | --- | --- | --- | --- | --- | --- | --- | --- | --- | --- | --- | --- | --- | --- | --- | --- | --- | --- | --- | --- | --- | --- | --- | --- | --- | --- | --- | --- | --- | --- | --- | --- | --- | --- | --- | --- | --- | --- | --- | --- | --- | --- | --- | --- | --- | --- | --- | --- | --- | --- | --- | --- | --- | --- | --- | --- | --- | --- | --- | --- | --- | --- | --- | --- | --- | --- | --- | --- | --- | --- | --- | --- | --- | --- | --- | --- | --- | --- | --- | --- | --- | --- | --- | --- | --- | --- | --- | --- | --- | --- | --- | --- | --- | --- | --- | --- | --- | --- | --- | --- | --- | --- | --- | --- | --- | --- | --- | --- | --- | --- | --- | --- | --- | --- | --- | --- | --- | --- | --- | --- | --- | --- | --- | --- | --- | --- | --- | --- | --- | --- | --- | --- | --- | --- | --- | --- | --- | --- | --- | --- | --- | --- | --- | --- | --- | --- | --- | --- | --- | --- | --- | --- | --- | --- | --- | --- | --- | --- | --- | --- | --- | --- | --- | --- | --- | --- | --- | --- | --- | --- | --- | --- | --- | --- | --- | --- | --- | --- | --- | --- | --- | --- | --- | --- | --- | --- | --- | --- | --- | --- | --- | --- | --- | --- | --- | --- | --- | --- | --- | --- | --- | --- | --- | --- | --- |

| **Supplementary Table 16. KEGG enrichment of expanded gene families in *A.edgeworthii.* Related to Figure 2.** | | | | |
| --- | --- | --- | --- | --- |
| **Map ID** | **MapTitle** | **P-value** | **Adjusted P-value** | **Gene Number** |
| map00195 | Photosynthesis | 1.44E-21 | 1.59E-19 | 28 |
| map00750 | Vitamin B6 metabolism | 2.82E-08 | 1.57E-06 | 10 |
| map00950 | Isoquinoline alkaloid biosynthesis | 2.66E-06 | 9.85E-05 | 12 |
| map00460 | Cyanoamino acid metabolism | 9.45E-06 | 0.000209752 | 13 |
| map00350 | Tyrosine metabolism | 0.00011792 | 0.002181612 | 12 |
| map03018 | RNA degradation | 0.00023239 | 0.0036851 | 19 |
| map00130 | Ubiquinone and other terpenoid-quinone biosynthesis | 0.00101281 | 0.014052681 | 8 |
| map04120 | Ubiquitin mediated proteolysis | 0.00152396 | 0.018332606 | 15 |
| map00440 | Phosphonate and phosphinate metabolism | 0.00165159 | 0.018332606 | 4 |
| map00940 | Phenylpropanoid biosynthesis | 0.00298954 | 0.030167177 | 15 |
| map00190 | Oxidative phosphorylation | 0.00780302 | 0.066625755 | 11 |
| map02010 | ABC transporters | 0.01109199 | 0.07695067 | 8 |
| map03440 | Homologous recombination | 0.01109199 | 0.07695067 | 8 |
| map03030 | DNA replication | 0.01216763 | 0.079447486 | 8 |
| map00966 | Glucosinolate biosynthesis | 0.01903686 | 0.117393967 | 3 |
| map03013 | RNA transport | 0.02288987 | 0.133725024 | 2 |
| map04626 | Plant-pathogen interaction | 0.02487427 | 0.138052182 | 22 |
| map01200 | Carbon metabolism | 0.02673276 | 0.141301732 | 4 |
| map00906 | Carotenoid biosynthesis | 0.03423428 | 0.172727497 | 5 |
| map04144 | Endocytosis | 0.0379089 | 0.18295165 | 4 |

**Supplementary Table 17. GO enrichment of contracted gene families in *A.edgeworthii.* Related to Figure 2.**

| **GO ID** | **GO Term** | **GO Class** | **P-value** | **Adjusted P-value** | **Gene Number** |
| --- | --- | --- | --- | --- | --- |
| GO:0005215 | transporter activity | MF | 1.09E-77 | 8.80E-75 | 215 |
| GO:0004713 | protein tyrosine kinase activity | MF | 7.06E-77 | 2.85E-74 | 205 |
| GO:0032559 | adenyl ribonucleotide binding | MF | 4.20E-76 | 1.13E-73 | 360 |
| GO:0000166 | nucleotide binding | MF | 1.82E-70 | 2.44E-68 | 424 |
| GO:0022857 | transmembrane transporter activity | MF | 1.29E-67 | 1.49E-65 | 170 |
| GO:0022804 | active transmembrane transporter activity | MF | 3.68E-67 | 3.71E-65 | 103 |
| GO:0006468 | protein phosphorylation | BP | 1.28E-64 | 1.03E-62 | 217 |
| GO:0004672 | protein kinase activity | MF | 3.37E-64 | 2.47E-62 | 218 |
| GO:0043492 | ATPase activity, coupled to movement of substances | MF | 7.75E-64 | 5.21E-62 | 65 |
| GO:0005524 | ATP binding | MF | 3.45E-63 | 2.14E-61 | 334 |
| GO:0042626 | ATPase activity, coupled to transmembrane movement of substances | MF | 6.53E-63 | 3.77E-61 | 64 |
| GO:0016773 | phosphotransferase activity, alcohol group as acceptor | MF | 8.85E-62 | 4.76E-60 | 238 |
| GO:0032550 | purine ribonucleoside binding | MF | 1.88E-61 | 8.44E-60 | 370 |
| GO:0032555 | purine ribonucleotide binding | MF | 1.88E-61 | 8.44E-60 | 370 |
| GO:0032553 | ribonucleotide binding | MF | 6.86E-61 | 2.91E-59 | 373 |
| GO:0016301 | kinase activity | MF | 1.03E-57 | 3.31E-56 | 236 |
| GO:0043168 | anion binding | MF | 1.25E-55 | 3.47E-54 | 404 |
| GO:0035639 | purine ribonucleoside triphosphate binding | MF | 2.96E-50 | 7.97E-49 | 344 |
| GO:0044765 | single-organism transport | BP | 3.69E-46 | 9.60E-45 | 220 |
| GO:0003824 | catalytic activity | MF | 4.03E-46 | 1.02E-44 | 883 |
| GO:0006464 | cellular protein modification process | BP | 3.37E-44 | 7.99E-43 | 228 |
| GO:0022891 | substrate-specific transmembrane transporter activity | MF | 1.08E-43 | 2.50E-42 | 123 |
| GO:0022892 | substrate-specific transporter activity | MF | 2.02E-43 | 4.54E-42 | 128 |
| GO:0016772 | transferase activity, transferring phosphorus-containing groups | MF | 5.32E-43 | 1.16E-41 | 246 |
| GO:0055085 | transmembrane transport | BP | 2.62E-39 | 5.57E-38 | 150 |
| GO:0019829 | cation-transporting ATPase activity | MF | 5.84E-38 | 1.15E-36 | 42 |
| GO:0016020 | membrane | CC | 1.07E-36 | 2.01E-35 | 326 |
| GO:0031224 | intrinsic component of membrane | CC | 1.47E-35 | 2.69E-34 | 170 |
| GO:0006796 | phosphate-containing compound metabolic process | BP | 5.37E-34 | 9.63E-33 | 241 |
| GO:0006810 | transport | BP | 7.11E-33 | 1.22E-31 | 260 |
| GO:0016021 | integral component of membrane | CC | 3.36E-32 | 5.54E-31 | 162 |
| GO:0016740 | transferase activity | MF | 4.78E-30 | 7.57E-29 | 364 |
| GO:0015075 | ion transmembrane transporter activity | MF | 1.13E-29 | 1.75E-28 | 98 |
| GO:0044425 | membrane part | CC | 1.53E-26 | 2.24E-25 | 185 |
| GO:0016887 | ATPase activity | MF | 5.55E-26 | 7.72E-25 | 86 |
| GO:0043167 | ion binding | MF | 1.61E-25 | 2.17E-24 | 555 |
| GO:0043086 | negative regulation of catalytic activity | BP | 3.90E-24 | 5.00E-23 | 34 |
| GO:0017111 | nucleoside-triphosphatase activity | MF | 2.23E-23 | 2.81E-22 | 151 |
| GO:0034637 | cellular carbohydrate biosynthetic process | BP | 9.98E-23 | 1.22E-21 | 32 |
| GO:0042802 | identical protein binding | MF | 1.56E-22 | 1.88E-21 | 34 |
| GO:0008324 | cation transmembrane transporter activity | MF | 9.16E-21 | 1.03E-19 | 74 |
| GO:0042623 | ATPase activity, coupled | MF | 2.91E-20 | 3.18E-19 | 68 |
| GO:0004553 | hydrolase activity, hydrolyzing O-glycosyl compounds | MF | 2.29E-19 | 2.44E-18 | 88 |
| GO:0042221 | response to chemical | BP | 2.29E-19 | 2.44E-18 | 37 |
| GO:0016798 | hydrolase activity, acting on glycosyl bonds | MF | 3.48E-19 | 3.65E-18 | 91 |
| GO:0006811 | ion transport | BP | 2.25E-18 | 2.32E-17 | 98 |
| GO:0004252 | serine-type endopeptidase activity | MF | 4.23E-18 | 4.26E-17 | 35 |
| GO:0015297 | antiporter activity | MF | 4.87E-18 | 4.85E-17 | 39 |
| GO:0044699 | single-organism process | BP | 2.26E-17 | 2.20E-16 | 506 |
| GO:0005975 | carbohydrate metabolic process | BP | 5.03E-17 | 4.84E-16 | 127 |
| GO:0019538 | protein metabolic process | BP | 1.38E-16 | 1.28E-15 | 297 |
| GO:0008272 | sulfate transport | BP | 2.12E-16 | 1.86E-15 | 16 |
| GO:0015116 | sulfate transmembrane transporter activity | MF | 2.12E-16 | 1.86E-15 | 16 |
| GO:0016787 | hydrolase activity | MF | 7.19E-16 | 6.11E-15 | 337 |
| GO:0004012 | phospholipid-translocating ATPase activity | MF | 2.28E-15 | 1.86E-14 | 15 |
| GO:0015914 | phospholipid transport | BP | 2.28E-15 | 1.86E-14 | 15 |
| GO:0006820 | anion transport | BP | 3.51E-15 | 2.80E-14 | 35 |
| GO:0003774 | motor activity | MF | 1.33E-14 | 1.05E-13 | 29 |
| GO:0003843 | 1,3-beta-D-glucan synthase activity | MF | 1.78E-14 | 1.39E-13 | 15 |
| GO:1901363 | heterocyclic compound binding | MF | 2.78E-14 | 2.16E-13 | 514 |
| GO:0097159 | organic cyclic compound binding | MF | 2.92E-14 | 2.22E-13 | 514 |
| GO:0043531 | ADP binding | MF | 3.28E-14 | 2.48E-13 | 26 |
| GO:0044267 | cellular protein metabolic process | BP | 9.82E-14 | 7.14E-13 | 244 |
| GO:0016459 | myosin complex | CC | 1.80E-13 | 1.25E-12 | 16 |
| GO:0033692 | cellular polysaccharide biosynthetic process | BP | 1.87E-13 | 1.28E-12 | 24 |
| GO:0006855 | drug transmembrane transport | BP | 2.59E-13 | 1.70E-12 | 23 |
| GO:0015238 | drug transmembrane transporter activity | MF | 2.59E-13 | 1.70E-12 | 23 |
| GO:0035251 | UDP-glucosyltransferase activity | MF | 1.02E-12 | 6.54E-12 | 24 |
| GO:0000148 | 1,3-beta-D-glucan synthase complex | CC | 2.72E-12 | 1.69E-11 | 12 |
| GO:0006075 | (1->3)-beta-D-glucan biosynthetic process | BP | 2.72E-12 | 1.69E-11 | 12 |
| GO:0006812 | cation transport | BP | 2.74E-12 | 1.69E-11 | 74 |
| GO:0071944 | cell periphery | CC | 3.81E-12 | 2.30E-11 | 42 |
| GO:0071805 | potassium ion transmembrane transport | BP | 5.03E-12 | 2.96E-11 | 15 |
| GO:0051274 | beta-glucan biosynthetic process | BP | 6.10E-12 | 3.52E-11 | 21 |
| GO:0044430 | cytoskeletal part | CC | 3.10E-11 | 1.75E-10 | 36 |
| GO:0044262 | cellular carbohydrate metabolic process | BP | 4.02E-11 | 2.25E-10 | 38 |
| GO:0044264 | cellular polysaccharide metabolic process | BP | 5.42E-11 | 3.02E-10 | 25 |
| GO:0016307 | phosphatidylinositol phosphate kinase activity | MF | 1.12E-10 | 6.04E-10 | 13 |
| GO:0044763 | single-organism cellular process | BP | 4.61E-10 | 2.43E-09 | 317 |
| GO:0006952 | defense response | BP | 5.57E-10 | 2.85E-09 | 28 |
| GO:0071555 | cell wall organization | BP | 1.72E-09 | 8.40E-09 | 29 |
| GO:0030246 | carbohydrate binding | MF | 3.29E-09 | 1.60E-08 | 34 |
| GO:0044459 | plasma membrane part | CC | 3.62E-09 | 1.74E-08 | 15 |
| GO:0042545 | cell wall modification | BP | 4.56E-09 | 2.15E-08 | 21 |
| GO:0006073 | cellular glucan metabolic process | BP | 5.88E-09 | 2.74E-08 | 22 |
| GO:0015079 | potassium ion transmembrane transporter activity | MF | 6.65E-09 | 3.07E-08 | 15 |
| GO:0005886 | plasma membrane | CC | 1.08E-08 | 4.89E-08 | 16 |
| GO:0000159 | protein phosphatase type 2A complex | CC | 2.10E-08 | 9.28E-08 | 10 |
| GO:0008601 | protein phosphatase type 2A regulator activity | MF | 2.10E-08 | 9.28E-08 | 10 |
| GO:0050660 | flavin adenine dinucleotide binding | MF | 3.04E-08 | 1.30E-07 | 22 |
| GO:0004435 | phosphatidylinositol phospholipase C activity | MF | 4.27E-08 | 1.79E-07 | 7 |
| GO:0009725 | response to hormone | BP | 6.55E-08 | 2.68E-07 | 14 |
| GO:0071554 | cell wall organization or biogenesis | BP | 1.12E-07 | 4.56E-07 | 35 |
| GO:0008236 | serine-type peptidase activity | MF | 1.75E-07 | 6.98E-07 | 37 |
| GO:0009987 | cellular process | BP | 2.00E-07 | 7.90E-07 | 636 |
| GO:0015711 | organic anion transport | BP | 2.25E-07 | 8.86E-07 | 19 |
| GO:0005507 | copper ion binding | MF | 1.04E-06 | 3.99E-06 | 24 |
| GO:0008081 | phosphoric diester hydrolase activity | MF | 2.17E-06 | 8.26E-06 | 11 |
| GO:0015299 | solute:proton antiporter activity | MF | 2.69E-06 | 1.01E-05 | 16 |
| GO:0008762 | UDP-N-acetylmuramate dehydrogenase activity | MF | 3.23E-06 | 1.21E-05 | 8 |
| GO:0015077 | monovalent inorganic cation transmembrane transporter activity | MF | 3.57E-06 | 1.33E-05 | 31 |
| GO:0030570 | pectate lyase activity | MF | 5.46E-06 | 2.01E-05 | 5 |
| GO:0006869 | lipid transport | BP | 6.44E-06 | 2.35E-05 | 16 |
| GO:0010215 | cellulose microfibril organization | BP | 6.94E-06 | 2.44E-05 | 8 |
| GO:0016049 | cell growth | BP | 6.94E-06 | 2.44E-05 | 8 |
| GO:0048544 | recognition of pollen | BP | 1.31E-05 | 4.46E-05 | 13 |
| GO:0031225 | anchored component of membrane | CC | 1.37E-05 | 4.67E-05 | 8 |
| GO:0005618 | cell wall | CC | 1.45E-05 | 4.91E-05 | 23 |
| GO:0004499 | N,N-dimethylaniline monooxygenase activity | MF | 2.26E-05 | 7.60E-05 | 9 |
| GO:0009690 | cytokinin metabolic process | BP | 3.04E-05 | 9.84E-05 | 5 |
| GO:0019139 | cytokinin dehydrogenase activity | MF | 3.04E-05 | 9.84E-05 | 5 |
| GO:0052861 | glucan endo-1,3-beta-glucanase activity, C-3 substituted reducing group | MF | 3.04E-05 | 9.84E-05 | 5 |
| GO:0052862 | glucan endo-1,4-beta-glucanase activity, C-3 substituted reducing group | MF | 3.04E-05 | 9.84E-05 | 5 |
| GO:0008661 | 1-deoxy-D-xylulose-5-phosphate synthase activity | MF | 3.04E-05 | 9.84E-05 | 5 |
| GO:0044723 | single-organism carbohydrate metabolic process | BP | 3.06E-05 | 9.88E-05 | 45 |
| GO:0005200 | structural constituent of cytoskeleton | MF | 4.41E-05 | 0.00014172 | 8 |
| GO:0005516 | calmodulin binding | MF | 7.32E-05 | 0.00023158 | 8 |
| GO:0016760 | cellulose synthase (UDP-forming) activity | MF | 8.60E-05 | 0.00026797 | 9 |
| GO:0004970 | ionotropic glutamate receptor activity | MF | 9.84E-05 | 0.00029755 | 5 |
| GO:0005234 | extracellular-glutamate-gated ion channel activity | MF | 9.84E-05 | 0.00029755 | 5 |
| GO:0050662 | coenzyme binding | MF | 0.00010305 | 0.00031029 | 40 |
| GO:0004175 | endopeptidase activity | MF | 0.00022063 | 0.00065458 | 43 |
| GO:0004849 | uridine kinase activity | MF | 0.00024326 | 0.00071386 | 5 |
| GO:0005874 | microtubule | CC | 0.00026713 | 0.00078107 | 8 |
| GO:0004888 | transmembrane signaling receptor activity | MF | 0.00027882 | 0.00081229 | 6 |
| GO:0004372 | glycine hydroxymethyltransferase activity | MF | 0.00028679 | 0.00081782 | 4 |
| GO:0006606 | protein import into nucleus | BP | 0.00028679 | 0.00081782 | 4 |
| GO:0004351 | glutamate decarboxylase activity | MF | 0.00028679 | 0.00081782 | 4 |
| GO:0000287 | magnesium ion binding | MF | 0.00033039 | 0.00093552 | 18 |
| GO:0048037 | cofactor binding | MF | 0.00034369 | 0.00096979 | 49 |
| GO:0016758 | transferase activity, transferring hexosyl groups | MF | 0.00035166 | 0.00098537 | 47 |
| GO:0008152 | metabolic process | BP | 0.00038414 | 0.00106896 | 720 |
| GO:0005992 | trehalose biosynthetic process | BP | 0.00054802 | 0.00151455 | 8 |
| GO:0030244 | cellulose biosynthetic process | BP | 0.00064669 | 0.00176908 | 9 |
| GO:0004420 | hydroxymethylglutaryl-CoA reductase (NADPH) activity | MF | 0.0006971 | 0.00190055 | 3 |
| GO:0005515 | protein binding | MF | 0.00092022 | 0.00243481 | 339 |
| GO:0016165 | linoleate 13S-lipoxygenase activity | MF | 0.00094047 | 0.00248025 | 5 |
| GO:0007017 | microtubule-based process | BP | 0.00095853 | 0.00251966 | 21 |
| GO:0015630 | microtubule cytoskeleton | CC | 0.00122418 | 0.00317656 | 20 |
| GO:0022607 | cellular component assembly | BP | 0.00122978 | 0.00318088 | 24 |
| GO:0016841 | ammonia-lyase activity | MF | 0.00173462 | 0.0044159 | 4 |
| GO:0008831 | dTDP-4-dehydrorhamnose reductase activity | MF | 0.00260322 | 0.006464 | 3 |
| GO:0045226 | extracellular polysaccharide biosynthetic process | BP | 0.00260322 | 0.006464 | 3 |
| GO:0050661 | NADP binding | MF | 0.00420965 | 0.01035728 | 9 |
| GO:0016614 | oxidoreductase activity, acting on CH-OH group of donors | MF | 0.00431966 | 0.01056353 | 21 |
| GO:0030599 | pectinesterase activity | MF | 0.00431966 | 0.01056353 | 21 |
| GO:0050664 | oxidoreductase activity, acting on NAD(P)H, oxygen as acceptor | MF | 0.00540094 | 0.01301063 | 4 |
| GO:0008889 | glycerophosphodiester phosphodiesterase activity | MF | 0.00540094 | 0.01301063 | 4 |
| GO:0003777 | microtubule motor activity | MF | 0.00571152 | 0.01359646 | 13 |
| GO:0007018 | microtubule-based movement | BP | 0.00571152 | 0.01359646 | 13 |
| GO:0051258 | protein polymerization | BP | 0.00573072 | 0.01360203 | 8 |
| GO:0008378 | galactosyltransferase activity | MF | 0.00621469 | 0.01470748 | 7 |
| GO:0004965 | G-protein coupled GABA receptor activity | MF | 0.00786716 | 0.01783369 | 2 |
| GO:0032065 | cortical protein anchoring | BP | 0.00786716 | 0.01783369 | 2 |
| GO:0006542 | glutamine biosynthetic process | BP | 0.00786716 | 0.01783369 | 2 |
| GO:0003855 | 3-dehydroquinate dehydratase activity | MF | 0.00786716 | 0.01783369 | 2 |
| GO:0006071 | glycerol metabolic process | BP | 0.00837459 | 0.01887792 | 4 |
| GO:0022890 | inorganic cation transmembrane transporter activity | MF | 0.0088469 | 0.01977687 | 32 |
| GO:0044238 | primary metabolic process | BP | 0.011007 | 0.02426952 | 527 |
| GO:0016114 | terpenoid biosynthetic process | BP | 0.01365624 | 0.02986608 | 5 |
| GO:0071704 | organic substance metabolic process | BP | 0.01395594 | 0.03043903 | 547 |
| GO:0005871 | kinesin complex | CC | 0.0143471 | 0.03120784 | 12 |
| GO:0005216 | ion channel activity | MF | 0.01659053 | 0.03570282 | 8 |
| GO:0050896 | response to stimulus | BP | 0.01847124 | 0.03892917 | 96 |
| GO:0033926 | glycopeptide alpha-N-acetylgalactosaminidase activity | MF | 0.01857216 | 0.03892917 | 3 |
| GO:0030130 | clathrin coat of trans-Golgi network vesicle | CC | 0.01857216 | 0.03892917 | 3 |
| GO:0004356 | glutamate-ammonia ligase activity | MF | 0.02220726 | 0.04525571 | 2 |
| GO:0006563 | L-serine metabolic process | BP | 0.02299661 | 0.04651195 | 4 |
| GO:0031047 | gene silencing by RNA | BP | 0.02299661 | 0.04651195 | 4 |
| GO:0006536 | glutamate metabolic process | BP | 0.02299661 | 0.04651195 | 4 |
| GO:0098662 | inorganic cation transmembrane transport | BP | 0.02423644 | 0.0478874 | 16 |
| GO:0005488 | binding | MF | 0.02427007 | 0.0478874 | 827 |
| GO:0046488 | phosphatidylinositol metabolic process | BP | 0.0259281 | 0.05090992 | 13 |
| GO:0015936 | coenzyme A metabolic process | BP | 0.02777986 | 0.05389026 | 3 |
| GO:0004857 | enzyme inhibitor activity | MF | 0.03272232 | 0.0616984 | 22 |
| GO:0016616 | oxidoreductase activity, acting on the CH-OH group of donors, NAD or NADP as acceptor | MF | 0.03321392 | 0.06233403 | 16 |
| GO:0016701 | oxidoreductase activity, acting on single donors with incorporation of molecular oxygen | MF | 0.03635405 | 0.06759843 | 7 |
| GO:0015743 | malate transport | BP | 0.03809033 | 0.06986114 | 4 |
| GO:0016702 | oxidoreductase activity, acting on single donors with incorporation of molecular oxygen, incorporation of two atoms of oxygen | MF | 0.03894839 | 0.07111165 | 6 |
| GO:0034622 | cellular macromolecular complex assembly | BP | 0.04268022 | 0.07688156 | 15 |
| GO:0016298 | lipase activity | MF | 0.04394203 | 0.07897822 | 11 |
| GO:0048519 | negative regulation of biological process | BP | 0.04516429 | 0.08099463 | 6 |
| GO:0005506 | iron ion binding | MF | 0.04627827 | 0.08280834 | 36 |
| GO:0004650 | polygalacturonase activity | MF | 0.04923131 | 0.08751028 | 9 |

**Supplementary Table 18. KEGG enrichment of contracted gene families in *A.edgeworthii.* Related to Figure 2.**

| **Map ID** | **MapTitle** | **P-value** | **Adjusted P-value** | **Gene Number** |
| --- | --- | --- | --- | --- |
| map02010 | ABC transporters | 1.46E-33 | 3.60E-31 | 45 |
| map00604 | Glycosphingolipid biosynthesis | 4.23E-18 | 2.08E-16 | 31 |
| map00531 | Glycosaminoglycan degradation | 7.29E-18 | 2.99E-16 | 34 |
| map04626 | Plant-pathogen interaction | 8.75E-18 | 3.07E-16 | 94 |
| map04144 | Endocytosis | 1.39E-13 | 2.62E-12 | 76 |
| map00600 | Sphingolipid metabolism | 8.94E-12 | 1.37E-10 | 34 |
| map00052 | Galactose metabolism | 1.46E-09 | 1.50E-08 | 39 |
| map00040 | Pentose and glucuronate interconversions | 2.74E-09 | 2.70E-08 | 46 |
| map00500 | Starch and sucrose metabolism | 3.85E-09 | 3.64E-08 | 84 |
| map04075 | Plant hormone signal transduction | 3.22E-07 | 2.83E-06 | 82 |
| map00511 | Other glycan degradation | 3.64E-07 | 3.09E-06 | 32 |
| map00410 | beta-Alanine metabolism | 5.92E-07 | 4.86E-06 | 22 |
| map04122 | Sulfur relay system | 1.17E-05 | 8.69E-05 | 10 |
| map00260 | Glycine, serine and threonine metabolism | 0.00012215 | 0.00077363 | 21 |
| map00380 | Tryptophan metabolism | 0.00012265 | 0.00077363 | 14 |
| map03010 | Ribosome | 0.00011711 | 0.00077363 | 11 |
| map00230 | Purine metabolism | 0.00014827 | 0.0009008 | 6 |
| map00360 | Phenylalanine metabolism | 0.00017315 | 0.00101417 | 17 |
| map03018 | RNA degradation | 0.00019939 | 0.00110935 | 7 |
| map04712 | Circadian rhythm - plant | 0.00028222 | 0.00147713 | 1 |
| map00565 | Ether lipid metabolism | 0.00043717 | 0.00218592 | 13 |
| map00903 | Limonene and pinene degradation | 0.00044429 | 0.00218592 | 10 |
| map00943 | Isoflavonoid biosynthesis | 0.0005195 | 0.00245151 | 13 |
| map04146 | Peroxisome | 0.0005823 | 0.00265268 | 1 |
| map04120 | Ubiquitin mediated proteolysis | 0.00060219 | 0.00269341 | 5 |
| map00730 | Thiamine metabolism | 0.00069036 | 0.00303267 | 7 |
| map00950 | Isoquinoline alkaloid biosynthesis | 0.00077868 | 0.00330268 | 16 |
| map04933 | AGE-RAGE signaling pathway in diabetic complications | 0.00076824 | 0.00330268 | 14 |
| map00562 | Inositol phosphate metabolism | 0.00131344 | 0.00529682 | 22 |
| map00960 | Tropane, piperidine and pyridine alkaloid biosynthesis | 0.00135405 | 0.00537252 | 13 |
| map00908 | Zeatin biosynthesis | 0.00217584 | 0.00787143 | 7 |
| map00270 | Cysteine and methionine metabolism | 0.00239705 | 0.00854601 | 2 |
| map01210 | 2-Oxocarboxylic acid metabolism | 0.00313162 | 0.01085039 | 1 |
| map00944 | Flavone and flavonol biosynthesis | 0.00437711 | 0.01435693 | 7 |
| map01212 | Fatty acid metabolism | 0.00622252 | 0.01987975 | 1 |
| map00480 | Glutathione metabolism | 0.00651483 | 0.02046683 | 1 |
| map04141 | Protein processing in endoplasmic reticulum | 0.00883769 | 0.02498933 | 17 |
| map00062 | Fatty acid elongation | 0.00951921 | 0.02601917 | 10 |
| map00240 | Pyrimidine metabolism | 0.01491726 | 0.03903878 | 10 |
| map00350 | Tyrosine metabolism | 0.01566734 | 0.04057016 | 18 |
| map04070 | Phosphatidylinositol signaling system | 0.01826321 | 0.046317 | 21 |
| map00430 | Taurine and hypotaurine metabolism | 0.01914535 | 0.04757329 | 5 |
| map03008 | Ribosome biogenesis in eukaryotes | 0.02128754 | 0.05134053 | 5 |
| map01200 | Carbon metabolism | 0.02237934 | 0.05344967 | 23 |
| map00051 | Fructose and mannose metabolism | 0.03186414 | 0.07257944 | 6 |
| map00340 | Histidine metabolism | 0.04834129 | 0.1061782 | 6 |
| map00053 | Ascorbate and aldarate metabolism | 0.04978853 | 0.10838919 | 9 |

**Supplementary Table 19. Details of the population locations of *A.edgeworthii* sampled in China. Related to Figure 3.**

| **Population code** | **Locations** | **Lat.(N)/Long.(E)** | **Number** |
| --- | --- | --- | --- |
| HL | Harbin, Hei long jiang | 45.80°, 126.53° | 10 |
| NM | Chifeng, Nei Mongol | 42.25°, 118.88° | 10 |
| SD | Taian, Shan dong | 36.20°, 117.08° | 10 |
| HB | Huanggang, Hu bei | 30.45°, 114.87°, | 10 |
| SC | Yaan, Si chuan | 29.98°, 103.01° | 10 |

| **Supplementary Table 20. Sequencing quality of 48 samples. Related to Figure 3.** | | | | | | | |
| --- | --- | --- | --- | --- | --- | --- | --- |
| **Sample** | **Raw bases(bp)** | **Clean bases(bp)** | **Effective rate(%)** | **Error rate(%)** | **Q20(%)** | **Q30(%)** | **GC content(%)** |
| HB-1 | 4660480500 | 4657252500 | 99.93 | 0.03 | 97.81 | 93.53 | 33.48 |
| HB-10 | 3967320600 | 3963840900 | 99.91 | 0.03 | 97.93 | 93.98 | 33.62 |
| HB-2 | 5094690300 | 5091121500 | 99.93 | 0.03 | 97.72 | 93.35 | 33.89 |
| HB-3 | 4439349900 | 4435665900 | 99.92 | 0.03 | 97.47 | 93 | 33.88 |
| HB-4 | 4511851800 | 4508152800 | 99.92 | 0.03 | 97.67 | 93.22 | 33.59 |
| HB-5 | 5566187100 | 5561897700 | 99.92 | 0.03 | 97.65 | 93.15 | 34 |
| HB-6 | 3551411700 | 3547908000 | 99.9 | 0.03 | 97.97 | 94.03 | 33.77 |
| HB-7 | 4126860000 | 4123533300 | 99.92 | 0.03 | 97.49 | 93.03 | 33.69 |
| HB-8 | 4032879900 | 4029384600 | 99.91 | 0.03 | 98.02 | 94.18 | 33.83 |
| HB-9 | 4520979900 | 4517123700 | 99.91 | 0.03 | 97.61 | 93.27 | 33.62 |
| HL-1 | 4994485200 | 4989019500 | 99.89 | 0.03 | 98.05 | 94.2 | 35.59 |
| HL-10 | 4131579300 | 4126278300 | 99.87 | 0.02 | 98.16 | 94.5 | 35.36 |
| HL-2 | 5117781600 | 5114126700 | 99.93 | 0.03 | 97.5 | 92.8 | 35.12 |
| HL-3 | 4190019600 | 4184757000 | 99.87 | 0.03 | 97.85 | 93.79 | 34.87 |
| HL-4 | 5184420900 | 5179088700 | 99.9 | 0.03 | 97.56 | 92.98 | 36.31 |
| HL-5 | 4411816200 | 4407321600 | 99.9 | 0.03 | 97.33 | 92.65 | 35.2 |
| HL-6 | 4384714800 | 4381144200 | 99.92 | 0.03 | 97.72 | 93.31 | 35.24 |
| HL-7 | 4551386100 | 4547031600 | 99.9 | 0.03 | 97.58 | 93.05 | 35.6 |
| HL-8 | 3639561000 | 3635354700 | 99.88 | 0.03 | 97.89 | 93.91 | 35.33 |
| HL-9 | 3826833300 | 3823037400 | 99.9 | 0.03 | 97.89 | 93.9 | 35.15 |
| NM-1 | 4452181800 | 4447228200 | 99.89 | 0.03 | 97.5 | 93 | 34.19 |
| NM-10 | 4078948200 | 4072806000 | 99.85 | 0.03 | 97.44 | 92.94 | 34.64 |
| NM-2 | 3903054900 | 3900380400 | 99.93 | 0.03 | 97.86 | 93.75 | 34.22 |
| NM-3 | 4081831800 | 4078889100 | 99.93 | 0.03 | 98.02 | 94.12 | 33.73 |
| NM-4 | 4767223500 | 4755867900 | 99.76 | 0.03 | 97.42 | 92.99 | 35.99 |
| NM-5 | 4025647500 | 4022301600 | 99.92 | 0.03 | 98.09 | 94.33 | 33.9 |
| NM-7 | 3718274700 | 3715208100 | 99.92 | 0.03 | 98.09 | 94.31 | 34 |
| NM-9 | 4300982400 | 4295430000 | 99.87 | 0.03 | 97.52 | 93.1 | 34.36 |
| SC-1 | 4863135000 | 4860331800 | 99.94 | 0.03 | 97.73 | 93.36 | 33.7 |
| SC-10 | 4749271800 | 4746462000 | 99.94 | 0.03 | 97.68 | 93.16 | 33.28 |
| SC-2 | 3927928800 | 3924031500 | 99.9 | 0.03 | 97.5 | 92.81 | 34.15 |
| SC-3 | 4824702300 | 4821769800 | 99.94 | 0.03 | 97.62 | 93.05 | 33.82 |
| SC-4 | 4785170400 | 4782023700 | 99.93 | 0.03 | 98.03 | 94.19 | 33.78 |
| SC-5 | 3770035800 | 3767407800 | 99.93 | 0.03 | 97.47 | 92.79 | 33.57 |
| SC-6 | 5035721400 | 5032289400 | 99.93 | 0.03 | 97.42 | 92.65 | 33.69 |
| SC-7 | 5085820200 | 5082354900 | 99.93 | 0.03 | 97.53 | 92.89 | 33.88 |
| SC-8 | 4019190300 | 4016635500 | 99.94 | 0.03 | 97.58 | 93.14 | 33.7 |
| SC-9 | 4578454500 | 4575483900 | 99.94 | 0.03 | 97.82 | 93.73 | 33.44 |
| SD-1 | 3539277600 | 3535845600 | 99.9 | 0.03 | 98.06 | 94.3 | 34.39 |
| SD-10 | 4540294200 | 4536243600 | 99.91 | 0.03 | 97.97 | 93.94 | 34.92 |
| SD-2 | 3619904100 | 3617239800 | 99.93 | 0.03 | 98.03 | 94.17 | 34.15 |
| SD-3 | 4343844900 | 4340865600 | 99.93 | 0.03 | 97.93 | 93.98 | 33.73 |
| SD-4 | 4285654800 | 4281719700 | 99.91 | 0.03 | 98.05 | 94.22 | 34.95 |
| SD-5 | 4273417500 | 4269854100 | 99.92 | 0.03 | 98.06 | 94.23 | 34.91 |
| SD-6 | 4588149600 | 4584728400 | 99.93 | 0.03 | 97.78 | 93.49 | 34.6 |
| SD-7 | 3861139500 | 3857233500 | 99.9 | 0.03 | 97.52 | 92.88 | 34.63 |
| SD-8 | 4522446000 | 4518572700 | 99.91 | 0.03 | 97.87 | 93.69 | 34.62 |
| SD-9 | 3717254700 | 3713004900 | 99.89 | 0.03 | 98 | 94.16 | 34.84 |

| **Supplementary Table 21. Sequence alignment of 48 samples. Related to Figure 3.** | | | | | | |
| --- | --- | --- | --- | --- | --- | --- |
| **Sample** | **Clean reads** | **mapped reads** | **mapping rate** | **Average depth** | **Coverage_1X** | **Coverage_4X** |
| HB-1 | 31048350 | 30006019 | 96.64% | 11.44 | 88.70% | 80.61% |
| HB-10 | 26425606 | 25472392 | 96.39% | 9.98 | 88.36% | 78.65% |
| HB-2 | 33940810 | 32727287 | 96.42% | 12.38 | 88.72% | 80.78% |
| HB-3 | 29571106 | 28509077 | 96.41% | 11.31 | 89.78% | 80.31% |
| HB-4 | 30054352 | 29019780 | 96.56% | 10.93 | 88.44% | 79.53% |
| HB-5 | 37079318 | 35781260 | 96.50% | 13.16 | 88.84% | 81.15% |
| HB-6 | 23652720 | 22833604 | 96.54% | 8.82 | 87.81% | 75.48% |
| HB-7 | 27490222 | 26479838 | 96.32% | 10.71 | 88.37% | 79.06% |
| HB-8 | 26862564 | 25912337 | 96.46% | 10.25 | 88.16% | 77.96% |
| HB-9 | 30114158 | 29064943 | 96.52% | 11.74 | 88.68% | 80.48% |
| HL-1 | 33260130 | 32519106 | 97.77% | 12.04 | 90.20% | 81.74% |
| HL-10 | 27508522 | 26870025 | 97.68% | 10.14 | 90.24% | 80.27% |
| HL-2 | 34094178 | 33474673 | 98.18% | 12 | 91.32% | 85.49% |
| HL-3 | 27898380 | 27288984 | 97.82% | 10.4 | 91.71% | 82.71% |
| HL-4 | 34527258 | 29739871 | 86.13% | 10.83 | 90.36% | 80.80% |
| HL-5 | 29382144 | 28721869 | 97.75% | 11.28 | 90.71% | 82.28% |
| HL-6 | 29207628 | 28517047 | 97.64% | 10.63 | 90.14% | 80.04% |
| HL-7 | 30313544 | 29613418 | 97.69% | 10.95 | 89.65% | 78.91% |
| HL-8 | 24235698 | 23730744 | 97.92% | 9.11 | 89.87% | 78.82% |
| HL-9 | 25486916 | 24975595 | 97.99% | 9.8 | 90.15% | 79.51% |
| NM-1 | 29648188 | 27423667 | 92.50% | 11 | 88.19% | 78.45% |
| NM-10 | 27152040 | 17444802 | 64.25% | 6.66 | 85.68% | 61.85% |
| NM-2 | 26002536 | 25070016 | 96.41% | 9.73 | 87.66% | 75.96% |
| NM-3 | 27192594 | 26171123 | 96.24% | 10.18 | 87.56% | 76.96% |
| NM-4 | 31705786 | 13442749 | 42.40% | 4.72 | 79.89% | 38.70% |
| NM-5 | 26815344 | 25868804 | 96.47% | 10.06 | 87.86% | 77.11% |
| NM-7 | 24768054 | 23839943 | 96.25% | 9.3 | 87.51% | 74.54% |
| NM-9 | 28636200 | 23767765 | 83.00% | 9.42 | 87.67% | 74.85% |
| SC-1 | 32402212 | 31228832 | 96.38% | 11.84 | 88.53% | 80.47% |
| SC-10 | 31643080 | 30527175 | 96.47% | 11.78 | 88.78% | 81.17% |
| SC-2 | 26160210 | 25148092 | 96.13% | 10.27 | 87.86% | 76.85% |
| SC-3 | 32145132 | 30956322 | 96.30% | 11.82 | 89.50% | 81.03% |
| SC-4 | 31880158 | 30788699 | 96.58% | 11.87 | 88.49% | 80.50% |
| SC-5 | 25116052 | 24135460 | 96.10% | 9.8 | 87.96% | 77.87% |
| SC-6 | 33548596 | 32356896 | 96.45% | 12.36 | 88.71% | 81.14% |
| SC-7 | 33882366 | 32540310 | 96.04% | 12.39 | 88.55% | 80.37% |
| SC-8 | 26777570 | 25866447 | 96.60% | 10.24 | 88.19% | 78.30% |
| SC-9 | 30503226 | 29410813 | 96.42% | 11.6 | 88.57% | 80.73% |
| SD-1 | 23572304 | 22975630 | 97.47% | 9.03 | 90.52% | 78.21% |
| SD-10 | 30241624 | 29361580 | 97.09% | 11.13 | 90.38% | 81.28% |
| SD-2 | 24114932 | 23505483 | 97.47% | 9.04 | 90.54% | 79.22% |
| SD-3 | 28939104 | 28208982 | 97.48% | 10.94 | 91.02% | 83.91% |
| SD-4 | 28544798 | 27727970 | 97.14% | 10.57 | 90.24% | 80.28% |
| SD-5 | 28465694 | 27499194 | 96.60% | 10.63 | 89.88% | 79.63% |
| SD-6 | 30564856 | 29762551 | 97.38% | 11.31 | 90.51% | 81.94% |
| SD-7 | 25714890 | 24977292 | 97.13% | 10.03 | 90.21% | 79.69% |
| SD-8 | 30123818 | 29348421 | 97.43% | 11.23 | 90.46% | 81.76% |
| SD-9 | 24753366 | 24126116 | 0.9747 | 9.39 | 90.69% | 78.21% |

| **Supplementary Table 22. Statistics of SNP annotation. Related to Figure 3.** | | |
| --- | --- | --- |
| **Category** | | **Number of SNPs** |
| Total | | 1,565,692 |
| Upstream | | 98,776 |
| Exonic | Stop gain | 872 |
|  | Stop loss | 143 |
|  | Synonymous | 66,715 |
|  | Non-synonymous | 64,763 |
| Intronic | | 292,621 |
| Splicing | | 416 |
| Downstream | | 94,553 |
| Upstream/Downstream | | 16,488 |
| Intergenic | | 876,006 |
| ts | | 1,142,724 |
| tv | | 422,968 |
| ts/tv | | 2.70 |

| **Supplementary Table 23. KEGG enrichment of DEGs in 4 modules associated with flower morphs. Related to Figure 4.** | | | | |  |
| --- | --- | --- | --- | --- | --- |
| **Model** | **Map ID** | **KEGG** | **P value** | **Adjusted Pvalue** | **Number** |
| yellow module | map04145 | Phagosome | 1.03E-05 | 0.000931758 | 14 |
|  | map00520 | Amino sugar and nucleotide sugar metabolism | 5.47E-05 | 0.002625738 | 16 |
|  | map00260 | Glycine, serine and threonine metabolism | 0.00161254 | 0.04210513 | 9 |
|  | map01200 | Carbon metabolism | 0.00530748 | 0.095942815 | 20 |
|  | map01230 | Biosynthesis of amino acids | 0.00634665 | 0.099430802 | 19 |
|  | map04144 | Endocytosis | 0.00634665 | 0.099430802 | 19 |
|  | map03010 | Ribosome | 0.00808073 | 0.1186857 | 1 |
|  | map00020 | Citrate cycle (TCA cycle) | 0.00860249 | 0.118916835 | 6 |
|  | map00630 | Glyoxylate and dicarboxylate metabolism | 0.00938823 | 0.122568577 | 7 |
|  | map00300 | Lysine biosynthesis | 0.01258861 | 0.15570125 | 3 |
|  | map01210 | 2-Oxocarboxylic acid metabolism | 0.01369268 | 0.158975966 | 8 |
|  | map00190 | Oxidative phosphorylation | 0.03971163 | 0.341771366 | 9 |
|  | map04075 | Plant hormone signal transduction | 0.04181785 | 0.341771366 | 22 |
|  | map00053 | Ascorbate and aldarate metabolism | 0.04797069 | 0.37577043 | 4 |
|  | map03040 | Spliceosome | 0.05378637 | 0.401790722 | 2 |
|  | map00261 | Monobactam biosynthesis | 0.05477709 | 0.401790722 | 2 |
|  | map00270 | Cysteine and methionine metabolism | 0.05735734 | 0.401790722 | 8 |
|  | map00330 | Arginine and proline metabolism | 0.07707481 | 0.48268013 | 5 |
|  | map00230 | Purine metabolism | 0.0780504 | 0.48268013 | 2 |
|  | map00410 | beta-Alanine metabolism | 0.08490679 | 0.484576775 | 5 |
|  | map00640 | Propanoate metabolism | 0.11607184 | 0.55920353 | 3 |
|  | map00770 | Pantothenate and CoA biosynthesis | 0.12097153 | 0.55920353 | 3 |
|  | map00240 | Pyrimidine metabolism | 0.13025259 | 0.57753506 | 2 |
|  | map04141 | Protein processing in endoplasmic reticulum | 0.14082944 | 0.590980689 | 14 |
|  | map03018 | RNA degradation | 0.14417577 | 0.594408856 | 3 |
|  | map00860 | Porphyrin and chlorophyll metabolism | 0.16092991 | 0.607929622 | 4 |
|  | map00670 | One carbon pool by folate | 0.16107657 | 0.607929622 | 2 |
|  | map00010 | Glycolysis / Gluconeogenesis | 0.17949005 | 0.620296509 | 9 |
|  | map03008 | Ribosome biogenesis in eukaryotes | 0.19361281 | 0.643545482 | 1 |
|  | map00051 | Fructose and mannose metabolism | 0.20059235 | 0.643545482 | 7 |
|  | map00290 | Valine, leucine and isoleucine biosynthesis | 0.20160493 | 0.643545482 | 2 |
|  | map00710 | Carbon fixation in photosynthetic organisms | 0.20664506 | 0.643545482 | 7 |
|  | map03060 | Protein export | 0.20792448 | 0.643545482 | 3 |
|  | map04712 | Circadian rhythm - plant | 0.21086384 | 0.643545482 | 7 |
|  | map00052 | Galactose metabolism | 0.22981391 | 0.675078354 | 7 |
|  | map04130 | SNARE interactions in vesicular transport | 0.23749817 | 0.684103821 | 3 |
|  | map00660 | C5-Branched dibasic acid metabolism | 0.23870857 | 0.684103821 | 1 |
|  | map00071 | Fatty acid degradation | 0.24952779 | 0.706494353 | 3 |
|  | map00970 | Aminoacyl-tRNA biosynthesis | 0.26736658 | 0.739189963 | 1 |
|  | map00564 | Glycerophospholipid metabolism | 0.33756284 | 0.867679614 | 3 |
|  | map00940 | Phenylpropanoid biosynthesis | 0.35025824 | 0.867679614 | 9 |
|  | map02010 | ABC transporters | 0.37478468 | 0.875990685 | 1 |
|  | map00730 | Thiamine metabolism | 0.37964736 | 0.875990685 | 1 |
|  | map03420 | Nucleotide excision repair | 0.38505902 | 0.875990685 | 1 |
|  | map00620 | Pyruvate metabolism | 0.39532956 | 0.884785203 | 5 |
|  | map00130 | Ubiquinone and other terpenoid-quinone biosynthesis | 0.43423431 | 0.919525556 | 3 |
|  | map04626 | Plant-pathogen interaction | 0.43499823 | 0.919525556 | 9 |
|  | map04122 | Sulfur relay system | 0.446376 | 0.919525556 | 1 |
|  | map00280 | Valine, leucine and isoleucine degradation | 0.45449549 | 0.919525556 | 3 |
|  | map00450 | Selenocompound metabolism | 0.45883742 | 0.919525556 | 1 |
|  | map00360 | Phenylalanine metabolism | 0.46095627 | 0.919525556 | 3 |
|  | map00905 | Brassinosteroid biosynthesis | 0.47101932 | 0.919525556 | 1 |
|  | map00944 | Flavone and flavonol biosynthesis | 0.47101932 | 0.919525556 | 1 |
|  | map00040 | Pentose and glucuronate interconversions | 0.49620327 | 0.919525556 | 8 |
|  | map00900 | Terpenoid backbone biosynthesis | 0.5159113 | 0.919525556 | 3 |
|  | map00790 | Folate biosynthesis | 0.51707466 | 0.919525556 | 1 |
|  | map00941 | Flavonoid biosynthesis | 0.52517052 | 0.919525556 | 1 |
|  | map03440 | Homologous recombination | 0.52925562 | 0.919525556 | 1 |
|  | map00740 | Riboflavin metabolism | 0.53858121 | 0.919525556 | 1 |
|  | map00350 | Tyrosine metabolism | 0.54421435 | 0.919525556 | 4 |
|  | map00340 | Histidine metabolism | 0.54897386 | 0.919525556 | 1 |
|  | map00500 | Starch and sucrose metabolism | 0.5678938 | 0.919525556 | 16 |
|  | map00650 | Butanoate metabolism | 0.56906459 | 0.919525556 | 1 |
|  | map00591 | Linoleic acid metabolism | 0.58826343 | 0.919525556 | 1 |
|  | map00750 | Vitamin B6 metabolism | 0.58826343 | 0.919525556 | 1 |
|  | map04070 | Phosphatidylinositol signaling system | 0.59103075 | 0.919525556 | 2 |
|  | map00960 | Tropane, piperidine and pyridine alkaloid biosynthesis | 0.67168438 | 0.992741059 | 2 |
|  | map00906 | Carotenoid biosynthesis | 0.6773352 | 0.994836072 | 2 |
| turquoise module | map00280 | Valine, leucine and isoleucine degradation | 3.37E-09 | 1.03E-06 | 19 |
|  | map04146 | Peroxisome | 1.91E-06 | 0.00029233 | 26 |
|  | map03010 | Ribosome | 0.00010432 | 0.010640495 | 6 |
|  | map04075 | Plant hormone signal transduction | 0.00202023 | 0.123637867 | 56 |
|  | map00250 | Alanine, aspartate and glutamate metabolism | 0.00985175 | 0.376829247 | 9 |
|  | map04130 | SNARE interactions in vesicular transport | 0.01343676 | 0.449447616 | 10 |
|  | map00071 | Fatty acid degradation | 0.02453572 | 0.483098812 | 10 |
|  | map03030 | DNA replication | 0.02526007 | 0.483098812 | 1 |
|  | map00030 | Pentose phosphate pathway | 0.02931981 | 0.49843673 | 16 |
|  | map00640 | Propanoate metabolism | 0.03733128 | 0.498566536 | 7 |
|  | map00940 | Phenylpropanoid biosynthesis | 0.03881777 | 0.498566536 | 8 |
|  | map04120 | Ubiquitin mediated proteolysis | 0.03910326 | 0.498566536 | 24 |
|  | map00040 | Pentose and glucuronate interconversions | 0.05916686 | 0.548469444 | 8 |
|  | map00052 | Galactose metabolism | 0.06059952 | 0.548469444 | 19 |
|  | map04122 | Sulfur relay system | 0.06651311 | 0.548469444 | 4 |
|  | map00290 | Valine, leucine and isoleucine biosynthesis | 0.06748544 | 0.548469444 | 5 |
|  | map00460 | Cyanoamino acid metabolism | 0.06811059 | 0.548469444 | 2 |
|  | map00261 | Monobactam biosynthesis | 0.07741047 | 0.590080492 | 3 |
|  | map00591 | Linoleic acid metabolism | 0.08099144 | 0.590080492 | 5 |
|  | map01040 | Biosynthesis of unsaturated fatty acids | 0.09582242 | 0.634934828 | 5 |
|  | map00010 | Glycolysis / Gluconeogenesis | 0.09874513 | 0.634934828 | 21 |
|  | map00600 | Sphingolipid metabolism | 0.11053315 | 0.650869088 | 14 |
|  | map00965 | Betalain biosynthesis | 0.11601372 | 0.650869088 | 1 |
|  | map01212 | Fatty acid metabolism | 0.12691488 | 0.650869088 | 12 |
|  | map04712 | Circadian rhythm - plant | 0.12911737 | 0.650869088 | 17 |
|  | map00510 | N-Glycan biosynthesis | 0.13009183 | 0.650869088 | 1 |
|  | map00770 | Pantothenate and CoA biosynthesis | 0.13742181 | 0.650869088 | 6 |
|  | map00500 | Starch and sucrose metabolism | 0.1394178 | 0.650869088 | 27 |
|  | map00061 | Fatty acid biosynthesis | 0.14465399 | 0.655913525 | 8 |
|  | map03440 | Homologous recombination | 0.15015392 | 0.665899985 | 3 |
|  | map00660 | C5-Branched dibasic acid metabolism | 0.15860059 | 0.687266185 | 2 |
|  | map01200 | Carbon metabolism | 0.16134268 | 0.687266185 | 36 |
|  | map00565 | Ether lipid metabolism | 0.16549973 | 0.687577901 | 6 |
|  | map00592 | alpha-Linolenic acid metabolism | 0.17292826 | 0.687577901 | 10 |
|  | map03022 | Basal transcription factors | 0.19310282 | 0.703445984 | 7 |
|  | map04144 | Endocytosis | 0.19825132 | 0.71370476 | 20 |
|  | map00920 | Sulfur metabolism | 0.20253277 | 0.720639861 | 5 |
|  | map03430 | Mismatch repair | 0.2055464 | 0.722956315 | 2 |
|  | map04141 | Protein processing in endoplasmic reticulum | 0.21074802 | 0.725791266 | 18 |
|  | map03008 | Ribosome biogenesis in eukaryotes | 0.21109615 | 0.725791266 | 15 |
|  | map00590 | Arachidonic acid metabolism | 0.22283165 | 0.749302023 | 5 |
|  | map00480 | Glutathione metabolism | 0.2401635 | 0.765501512 | 11 |
|  | map00904 | Diterpenoid biosynthesis | 0.24055454 | 0.765501512 | 2 |
|  | map00073 | Cutin, suberine and wax biosynthesis | 0.24152585 | 0.765501512 | 2 |
|  | map00190 | Oxidative phosphorylation | 0.24627032 | 0.765501512 | 7 |
|  | map01230 | Biosynthesis of amino acids | 0.25179679 | 0.770498189 | 33 |
|  | map00270 | Cysteine and methionine metabolism | 0.25770211 | 0.78076086 | 14 |
|  | map04070 | Phosphatidylinositol signaling system | 0.28040655 | 0.813467992 | 6 |
|  | map00903 | Limonene and pinene degradation | 0.28580095 | 0.813467992 | 4 |
|  | map03015 | mRNA surveillance pathway | 0.29595149 | 0.813467992 | 20 |
|  | map00240 | Pyrimidine metabolism | 0.3105849 | 0.813467992 | 12 |
|  | map04933 | AGE-RAGE signaling pathway in diabetic complications | 0.32619993 | 0.813467992 | 2 |
|  | map00945 | Stilbenoid, diarylheptanoid and gingerol biosynthesis | 0.33105329 | 0.813467992 | 2 |
|  | map03020 | RNA polymerase | 0.33105329 | 0.813467992 | 2 |
|  | map00130 | Ubiquinone and other terpenoid-quinone biosynthesis | 0.34262934 | 0.813467992 | 7 |
|  | map00232 | Caffeine metabolism | 0.35058745 | 0.813467992 | 1 |
|  | map00910 | Nitrogen metabolism | 0.36367749 | 0.813467992 | 4 |
|  | map03410 | Base excision repair | 0.36568072 | 0.813467992 | 3 |
|  | map00350 | Tyrosine metabolism | 0.37725211 | 0.81865024 | 5 |
|  | map00300 | Lysine biosynthesis | 0.38210844 | 0.81865024 | 2 |
|  | map00950 | Isoquinoline alkaloid biosynthesis | 0.38904974 | 0.826730694 | 3 |
|  | map00620 | Pyruvate metabolism | 0.39343552 | 0.828783529 | 12 |
|  | map03060 | Protein export | 0.44232039 | 0.884640782 | 2 |
|  | map00564 | Glycerophospholipid metabolism | 0.45472536 | 0.897715875 | 12 |
|  | map00650 | Butanoate metabolism | 0.48416454 | 0.919140089 | 3 |
|  | map03050 | Proteasome | 0.48771987 | 0.919140089 | 3 |
|  | map00942 | Anthocyanin biosynthesis | 0.49261103 | 0.919140089 | 1 |
|  | map00604 | Glycosphingolipid biosynthesis - ganglio series | 0.50117028 | 0.929443059 | 8 |
|  | map00710 | Carbon fixation in photosynthetic organisms | 0.50946989 | 0.93351967 | 14 |
|  | map00511 | Other glycan degradation | 0.54054157 | 0.972974819 | 13 |
|  | map00380 | Tryptophan metabolism | 0.59975039 | 0.974286866 | 5 |
|  | map00072 | Synthesis and degradation of ketone bodies | 0.60360699 | 0.974286866 | 1 |
|  | map00630 | Glyoxylate and dicarboxylate metabolism | 0.61354389 | 0.974286866 | 8 |
|  | map00906 | Carotenoid biosynthesis | 0.61955694 | 0.974286866 | 5 |
|  | map00100 | Steroid biosynthesis | 0.62416912 | 0.974286866 | 5 |
|  | map00860 | Porphyrin and chlorophyll metabolism | 0.62465935 | 0.974286866 | 4 |
|  | map00400 | Phenylalanine, tyrosine and tryptophan biosynthesis | 0.62770878 | 0.974286866 | 3 |
|  | map00900 | Terpenoid backbone biosynthesis | 0.63261076 | 0.974286866 | 8 |
|  | map00360 | Phenylalanine metabolism | 0.63843779 | 0.974286866 | 7 |
|  | map00520 | Amino sugar and nucleotide sugar metabolism | 0.64088423 | 0.974286866 | 18 |
|  | map00909 | Sesquiterpenoid and triterpenoid biosynthesis | 0.65226822 | 0.974286866 | 2 |
|  | map00051 | Fructose and mannose metabolism | 0.6531605 | 0.974286866 | 13 |
|  | map04145 | Phagosome | 0.67445417 | 0.974286866 | 11 |
|  | map00450 | Selenocompound metabolism | 0.67490254 | 0.974286866 | 2 |
|  | map00603 | Glycosphingolipid biosynthesis - globo and isoglobo series | 0.6830367 | 0.974286866 | 2 |
|  | map01210 | 2-Oxocarboxylic acid metabolism | 0.68566814 | 0.974286866 | 10 |
|  | map03420 | Nucleotide excision repair | 0.69474878 | 0.974286866 | 7 |
|  | map00514 | Other types of O-glycan biosynthesis | 0.69982963 | 0.974286866 | 2 |
|  | map00670 | One carbon pool by folate | 0.71700205 | 0.974286866 | 2 |
|  | map00790 | Folate biosynthesis | 0.71700205 | 0.974286866 | 2 |
|  | map00780 | Biotin metabolism | 0.72278455 | 0.974286866 | 1 |
|  | map00902 | Monoterpenoid biosynthesis | 0.73493056 | 0.974286866 | 3 |
|  | map00760 | Nicotinate and nicotinamide metabolism | 0.74996761 | 0.974286866 | 3 |
|  | map00062 | Fatty acid elongation | 0.77383143 | 0.974286866 | 2 |
|  | map00513 | Various types of N-glycan biosynthesis | 0.77383143 | 0.974286866 | 2 |
|  | map00943 | Isoflavonoid biosynthesis | 0.78185288 | 0.974286866 | 4 |
|  | map00053 | Ascorbate and aldarate metabolism | 0.78429838 | 0.974286866 | 4 |
|  | map00310 | Lysine degradation | 0.79821065 | 0.974286866 | 3 |
|  | map00960 | Tropane, piperidine and pyridine alkaloid biosynthesis | 0.79916995 | 0.974286866 | 3 |
|  | map00970 | Aminoacyl-tRNA biosynthesis | 0.8036399 | 0.975848444 | 10 |
|  | map00020 | Citrate cycle (TCA cycle) | 0.81111534 | 0.981032776 | 5 |
|  | map00531 | Glycosaminoglycan degradation | 0.82436574 | 0.989238885 | 8 |
|  | map00941 | Flavonoid biosynthesis | 0.82436574 | 0.989238885 | 8 |
|  | map00561 | Glycerolipid metabolism | 0.82987584 | 0.991960963 | 16 |
|  | map03018 | RNA degradation | 0.83397878 | 0.992986408 | 21 |
| blue module | map03010 | Ribosome | 1.87E-18 | 4.55E-16 | 48 |
|  | map00195 | Photosynthesis | 1.37E-07 | 1.66E-05 | 17 |
|  | map00230 | Purine metabolism | 0.00050139 | 0.030459316 | 23 |
|  | map00903 | Limonene and pinene degradation | 0.00205561 | 0.099902623 | 6 |
|  | map04626 | Plant-pathogen interaction | 0.00541879 | 0.182140144 | 7 |
|  | map00196 | Photosynthesis - antenna proteins | 0.00595963 | 0.182140144 | 3 |
|  | map00860 | Porphyrin and chlorophyll metabolism | 0.00617672 | 0.182140144 | 9 |
|  | map00620 | Pyruvate metabolism | 0.00674593 | 0.182140144 | 12 |
|  | map04075 | Plant hormone signal transduction | 0.01398715 | 0.286175873 | 34 |
|  | map00071 | Fatty acid degradation | 0.01946199 | 0.358302389 | 7 |
|  | map00053 | Ascorbate and aldarate metabolism | 0.02089815 | 0.358302389 | 6 |
|  | map04141 | Protein processing in endoplasmic reticulum | 0.02943174 | 0.420700773 | 6 |
|  | map00340 | Histidine metabolism | 0.03464522 | 0.460721568 | 4 |
|  | map00780 | Biotin metabolism | 0.03791947 | 0.460721568 | 4 |
|  | map00290 | Valine, leucine and isoleucine biosynthesis | 0.0413651 | 0.478653307 | 4 |
|  | map00100 | Steroid biosynthesis | 0.04511585 | 0.493556488 | 6 |
|  | map00511 | Other glycan degradation | 0.04996459 | 0.505891437 | 1 |
|  | map00760 | Nicotinate and nicotinamide metabolism | 0.07503868 | 0.596805475 | 4 |
|  | map00380 | Tryptophan metabolism | 0.08958554 | 0.596805475 | 5 |
|  | map00600 | Sphingolipid metabolism | 0.09085122 | 0.596805475 | 1 |
|  | map00240 | Pyrimidine metabolism | 0.12038521 | 0.68741286 | 15 |
|  | map00531 | Glycosaminoglycan degradation | 0.13286773 | 0.68741286 | 1 |
|  | map00941 | Flavonoid biosynthesis | 0.13286773 | 0.68741286 | 1 |
|  | map01040 | Biosynthesis of unsaturated fatty acids | 0.17807337 | 0.804623886 | 3 |
|  | map03020 | RNA polymerase | 0.20309326 | 0.809572312 | 5 |
|  | map00061 | Fatty acid biosynthesis | 0.20655755 | 0.809572312 | 5 |
|  | map01212 | Fatty acid metabolism | 0.2173307 | 0.825177516 | 7 |
|  | map00232 | Caffeine metabolism | 0.22399733 | 0.831703674 | 1 |
|  | map00280 | Valine, leucine and isoleucine degradation | 0.24942232 | 0.891317991 | 5 |
|  | map00604 | Glycosphingolipid biosynthesis - ganglio series | 0.26873771 | 0.902211921 | 1 |
|  | map00630 | Glyoxylate and dicarboxylate metabolism | 0.27846047 | 0.902211921 | 6 |
|  | map00052 | Galactose metabolism | 0.29343758 | 0.92604328 | 4 |
|  | map04070 | Phosphatidylinositol signaling system | 0.32795242 | 0.940708558 | 3 |
|  | map00942 | Anthocyanin biosynthesis | 0.32872617 | 0.940708558 | 1 |
|  | map00350 | Tyrosine metabolism | 0.33700784 | 0.940708558 | 2 |
|  | map00660 | C5-Branched dibasic acid metabolism | 0.35262543 | 0.940708558 | 1 |
|  | map00945 | Stilbenoid, diarylheptanoid and gingerol biosynthesis | 0.36006213 | 0.940708558 | 4 |
|  | map04144 | Endocytosis | 0.3676598 | 0.940708558 | 12 |
|  | map00073 | Cutin, suberine and wax biosynthesis | 0.37298478 | 0.940708558 | 1 |
|  | map00650 | Butanoate metabolism | 0.38104363 | 0.940708558 | 2 |
|  | map00360 | Phenylalanine metabolism | 0.38325164 | 0.940708558 | 1 |
|  | map00030 | Pentose phosphate pathway | 0.39361575 | 0.947016104 | 3 |
|  | map00051 | Fructose and mannose metabolism | 0.40534785 | 0.960247778 | 4 |
|  | map00940 | Phenylpropanoid biosynthesis | 0.44011536 | 0.960247778 | 7 |
|  | map03440 | Homologous recombination | 0.45154367 | 0.960536626 | 2 |
|  | map00261 | Monobactam biosynthesis | 0.45997236 | 0.960536626 | 1 |
|  | map00563 | Glycosylphosphatidylinositol (GPI)-anchor biosynthesis | 0.4624806 | 0.960536626 | 3 |
|  | map01210 | 2-Oxocarboxylic acid metabolism | 0.49250195 | 0.961916339 | 3 |
|  | map03022 | Basal transcription factors | 0.52064298 | 0.961916339 | 1 |
|  | map00510 | N-Glycan biosynthesis | 0.52203149 | 0.961916339 | 1 |
|  | map00520 | Amino sugar and nucleotide sugar metabolism | 0.53245507 | 0.961916339 | 7 |
|  | map00440 | Phosphonate and phosphinate metabolism | 0.53290937 | 0.961916339 | 1 |
|  | map00730 | Thiamine metabolism | 0.53290937 | 0.961916339 | 1 |
|  | map04120 | Ubiquitin mediated proteolysis | 0.53958795 | 0.964116707 | 7 |
|  | map00300 | Lysine biosynthesis | 0.54955244 | 0.974753602 | 1 |
|  | map00190 | Oxidative phosphorylation | 1.26E-43 | 3.45E-41 | 40 |
|  | map00020 | Citrate cycle (TCA cycle) | 2.91E-08 | 1.14E-06 | 15 |
|  | map01200 | Carbon metabolism | 2.18E-05 | 0.000742252 | 32 |
|  | map00620 | Pyruvate metabolism | 0.00039835 | 0.012083414 | 14 |
|  | map00330 | Arginine and proline metabolism | 0.00549057 | 0.099928278 | 9 |
|  | map00062 | Fatty acid elongation | 0.00929698 | 0.148023766 | 6 |
|  | map00010 | Glycolysis / Gluconeogenesis | 0.01490718 | 0.176941715 | 15 |
|  | map00740 | Riboflavin metabolism | 0.02350578 | 0.246810683 | 4 |
|  | map01210 | 2-Oxocarboxylic acid metabolism | 0.02543196 | 0.249469425 | 10 |
|  | map00340 | Histidine metabolism | 0.02587948 | 0.249469425 | 4 |
|  | map00270 | Cysteine and methionine metabolism | 0.02692398 | 0.249469425 | 11 |
|  | map00710 | Carbon fixation in photosynthetic organisms | 0.02777638 | 0.249469425 | 12 |
|  | map00440 | Phosphonate and phosphinate metabolism | 0.02924184 | 0.249469425 | 3 |
|  | map01230 | Biosynthesis of amino acids | 0.03152532 | 0.260586825 | 23 |
|  | map04626 | Plant-pathogen interaction | 0.04633082 | 0.341846325 | 9 |
|  | map00220 | Arginine biosynthesis | 0.05945885 | 0.394846304 | 5 |
|  | map00905 | Brassinosteroid biosynthesis | 0.06106429 | 0.394846304 | 3 |
|  | map01212 | Fatty acid metabolism | 0.06653088 | 0.394846304 | 8 |
|  | map03420 | Nucleotide excision repair | 0.0944781 | 0.468954939 | 1 |
|  | map03013 | RNA transport | 0.09705669 | 0.473151363 | 18 |
|  | map03018 | RNA degradation | 0.10425197 | 0.492918429 | 5 |
|  | map04145 | Phagosome | 0.11352716 | 0.516548587 | 9 |
|  | map00903 | Limonene and pinene degradation | 0.12468188 | 0.549002459 | 3 |
|  | map01040 | Biosynthesis of unsaturated fatty acids | 0.1470909 | 0.60075318 | 3 |
|  | map00630 | Glyoxylate and dicarboxylate metabolism | 0.15704414 | 0.630486033 | 6 |
|  | map00520 | Amino sugar and nucleotide sugar metabolism | 0.16321964 | 0.636556592 | 13 |
|  | map00909 | Sesquiterpenoid and triterpenoid biosynthesis | 0.18219814 | 0.681371117 | 2 |
|  | map03440 | Homologous recombination | 0.19105015 | 0.687218487 | 1 |
|  | map00920 | Sulfur metabolism | 0.19526856 | 0.687218487 | 3 |
|  | map00970 | Aminoacyl-tRNA biosynthesis | 0.2452071 | 0.731884536 | 2 |
|  | map00600 | Sphingolipid metabolism | 0.24534162 | 0.731884536 | 2 |
|  | map00640 | Propanoate metabolism | 0.24664241 | 0.731884536 | 3 |
|  | map04075 | Plant hormone signal transduction | 0.25658377 | 0.750029055 | 15 |
|  | map03040 | Spliceosome | 0.25825176 | 0.750029055 | 7 |
|  | map00195 | Photosynthesis | 0.266174 | 0.753726501 | 1 |
|  | map00670 | One carbon pool by folate | 0.27861797 | 0.753726501 | 2 |
|  | map00310 | Lysine degradation | 0.284373 | 0.753726501 | 4 |
|  | map00564 | Glycerophospholipid metabolism | 0.3169397 | 0.787665252 | 5 |
|  | map00071 | Fatty acid degradation | 0.31825595 | 0.787665252 | 4 |
|  | map00780 | Biotin metabolism | 0.32696339 | 0.787665252 | 2 |
|  | map00061 | Fatty acid biosynthesis | 0.32743196 | 0.787665252 | 4 |
|  | map03008 | Ribosome biogenesis in eukaryotes | 0.33765633 | 0.787665252 | 3 |
|  | map00500 | Starch and sucrose metabolism | 0.34161502 | 0.787665252 | 24 |
|  | map03050 | Proteasome | 0.34209816 | 0.787665252 | 4 |
|  | map00030 | Pentose phosphate pathway | 0.35118658 | 0.792346582 | 7 |
|  | map03410 | Base excision repair | 0.37484792 | 0.818667858 | 1 |
|  | map00604 | Glycosphingolipid biosynthesis - ganglio series | 0.382464 | 0.822147013 | 1 |
|  | map03010 | Ribosome | 0.38939073 | 0.826472299 | 10 |
|  | map00072 | Synthesis and degradation of ketone bodies | 0.39053087 | 0.826472299 | 1 |
|  | map04144 | Endocytosis | 0.40473608 | 0.843926793 | 11 |
|  | map00460 | Cyanoamino acid metabolism | 0.4427525 | 0.883411893 | 2 |
|  | map00053 | Ascorbate and aldarate metabolism | 0.45066927 | 0.883411893 | 3 |
|  | map00250 | Alanine, aspartate and glutamate metabolism | 0.45066927 | 0.883411893 | 3 |
|  | map00052 | Galactose metabolism | 0.45404749 | 0.883411893 | 9 |
|  | map00230 | Purine metabolism | 0.46338625 | 0.883411893 | 13 |
|  | map00380 | Tryptophan metabolism | 0.48027715 | 0.897618646 | 3 |
|  | map03015 | mRNA surveillance pathway | 0.4930671 | 0.905992266 | 6 |
|  | map00730 | Thiamine metabolism | 0.50012789 | 0.905992266 | 1 |
|  | map00510 | N-Glycan biosynthesis | 0.50769379 | 0.905992266 | 3 |
|  | map04141 | Protein processing in endoplasmic reticulum | 0.52112888 | 0.905992266 | 16 |
|  | map03020 | RNA polymerase | 0.52200163 | 0.905992266 | 1 |
|  | map00130 | Ubiquinone and other terpenoid-quinone biosynthesis | 0.52554674 | 0.905992266 | 1 |
|  | map00051 | Fructose and mannose metabolism | 0.52956196 | 0.905992266 | 4 |
|  | map00280 | Valine, leucine and isoleucine degradation | 0.53762178 | 0.905992266 | 4 |
|  | map04122 | Sulfur relay system | 0.57627629 | 0.922930713 | 1 |
|  | map00410 | beta-Alanine metabolism | 0.58195085 | 0.922930713 | 4 |
|  | map00900 | Terpenoid backbone biosynthesis | 0.58746527 | 0.922930713 | 2 |
|  | map00531 | Glycosaminoglycan degradation | 0.59280996 | 0.922930713 | 2 |
|  | map00511 | Other glycan degradation | 0.59500295 | 0.922930713 | 4 |
|  | map00940 | Phenylpropanoid biosynthesis | 0.6094747 | 0.926156541 | 7 |
|  | map04712 | Circadian rhythm - plant | 0.61275389 | 0.926156541 | 8 |
|  | map00562 | Inositol phosphate metabolism | 0.63481788 | 0.952226821 | 3 |
|  | map00760 | Nicotinate and nicotinamide metabolism | 0.65765982 | 0.961503237 | 2 |
|  | map00513 | Various types of N-glycan biosynthesis | 0.70380735 | 0.976023578 | 2 |
|  | map00563 | Glycosylphosphatidylinositol (GPI)-anchor biosynthesis | 0.71285758 | 0.976023578 | 2 |
|  | map04120 | Ubiquitin mediated proteolysis | 0.72535679 | 0.976023578 | 10 |
|  | map03060 | Protein export | 0.72950485 | 0.976023578 | 1 |
|  | map00906 | Carotenoid biosynthesis | 0.73200566 | 0.976023578 | 1 |
|  | map00400 | Phenylalanine, tyrosine and tryptophan biosynthesis | 0.73648666 | 0.976023578 | 3 |
|  | map04130 | SNARE interactions in vesicular transport | 0.73648666 | 0.976023578 | 3 |
|  | map00360 | Phenylalanine metabolism | 0.77083533 | 0.999364856 | 3 |
| brown moudle | map00190 | Oxidative phosphorylation | 1.26E-43 | 3.45E-41 | 40 |
|  | map00020 | Citrate cycle (TCA cycle) | 2.91E-08 | 1.14E-06 | 15 |
|  | map01200 | Carbon metabolism | 2.18E-05 | 0.000742252 | 32 |
|  | map00620 | Pyruvate metabolism | 0.00039835 | 0.012083414 | 14 |
|  | map00330 | Arginine and proline metabolism | 0.00549056 | 0.099928278 | 9 |
|  | map00062 | Fatty acid elongation | 0.00929698 | 0.148023766 | 6 |
|  | map00010 | Glycolysis / Gluconeogenesis | 0.01490718 | 0.176941715 | 15 |
|  | map00740 | Riboflavin metabolism | 0.02350578 | 0.246810683 | 4 |
|  | map01210 | 2-Oxocarboxylic acid metabolism | 0.02543196 | 0.249469425 | 10 |
|  | map00340 | Histidine metabolism | 0.02587948 | 0.249469425 | 4 |
|  | map00270 | Cysteine and methionine metabolism | 0.02692398 | 0.249469425 | 11 |
|  | map00710 | Carbon fixation in photosynthetic organisms | 0.02777638 | 0.249469425 | 12 |
|  | map00440 | Phosphonate and phosphinate metabolism | 0.02924184 | 0.249469425 | 3 |
|  | map01230 | Biosynthesis of amino acids | 0.03152532 | 0.260586825 | 23 |
|  | map04626 | Plant-pathogen interaction | 0.04633082 | 0.341846325 | 9 |
|  | map00220 | Arginine biosynthesis | 0.05945884 | 0.394846304 | 5 |
|  | map00905 | Brassinosteroid biosynthesis | 0.06106429 | 0.394846304 | 3 |
|  | map01212 | Fatty acid metabolism | 0.06653088 | 0.394846304 | 8 |
|  | map03420 | Nucleotide excision repair | 0.0944781 | 0.468954939 | 1 |
|  | map03013 | RNA transport | 0.09705669 | 0.473151363 | 18 |
|  | map03018 | RNA degradation | 0.10425197 | 0.492918429 | 5 |
|  | map04145 | Phagosome | 0.11352716 | 0.516548587 | 9 |
|  | map00903 | Limonene and pinene degradation | 0.12468188 | 0.549002459 | 3 |
|  | map01040 | Biosynthesis of unsaturated fatty acids | 0.1470909 | 0.60075318 | 3 |
|  | map00630 | Glyoxylate and dicarboxylate metabolism | 0.15704414 | 0.630486033 | 6 |
|  | map00520 | Amino sugar and nucleotide sugar metabolism | 0.16321964 | 0.636556592 | 13 |
|  | map00909 | Sesquiterpenoid and triterpenoid biosynthesis | 0.18219814 | 0.681371117 | 2 |
|  | map03440 | Homologous recombination | 0.19105015 | 0.687218487 | 1 |
|  | map00920 | Sulfur metabolism | 0.19526856 | 0.687218487 | 3 |
|  | map00970 | Aminoacyl-tRNA biosynthesis | 0.2452071 | 0.731884536 | 2 |
|  | map00600 | Sphingolipid metabolism | 0.24534162 | 0.731884536 | 2 |
|  | map00640 | Propanoate metabolism | 0.24664241 | 0.731884536 | 3 |
|  | map04075 | Plant hormone signal transduction | 0.25658377 | 0.750029055 | 15 |
|  | map03040 | Spliceosome | 0.25825176 | 0.750029055 | 7 |
|  | map00195 | Photosynthesis | 0.266174 | 0.753726501 | 1 |
|  | map00670 | One carbon pool by folate | 0.27861797 | 0.753726501 | 2 |
|  | map00310 | Lysine degradation | 0.284373 | 0.753726501 | 4 |
|  | map00564 | Glycerophospholipid metabolism | 0.31693969 | 0.787665252 | 5 |
|  | map00071 | Fatty acid degradation | 0.31825595 | 0.787665252 | 4 |
|  | map00780 | Biotin metabolism | 0.32696339 | 0.787665252 | 2 |
|  | map00061 | Fatty acid biosynthesis | 0.32743196 | 0.787665252 | 4 |
|  | map03008 | Ribosome biogenesis in eukaryotes | 0.33765633 | 0.787665252 | 3 |
|  | map00500 | Starch and sucrose metabolism | 0.34161502 | 0.787665252 | 24 |
|  | map03050 | Proteasome | 0.34209816 | 0.787665252 | 4 |
|  | map00030 | Pentose phosphate pathway | 0.35118658 | 0.792346582 | 7 |
|  | map03410 | Base excision repair | 0.37484792 | 0.818667858 | 1 |
|  | map00604 | Glycosphingolipid biosynthesis - ganglio series | 0.382464 | 0.822147013 | 1 |
|  | map03010 | Ribosome | 0.38939073 | 0.826472299 | 10 |
|  | map00072 | Synthesis and degradation of ketone bodies | 0.39053087 | 0.826472299 | 1 |
|  | map04144 | Endocytosis | 0.40473608 | 0.843926793 | 11 |
|  | map00460 | Cyanoamino acid metabolism | 0.4427525 | 0.883411893 | 2 |
|  | map00053 | Ascorbate and aldarate metabolism | 0.45066927 | 0.883411893 | 3 |
|  | map00250 | Alanine, aspartate and glutamate metabolism | 0.45066927 | 0.883411893 | 3 |
|  | map00052 | Galactose metabolism | 0.45404749 | 0.883411893 | 9 |
|  | map00230 | Purine metabolism | 0.46338624 | 0.883411893 | 13 |
|  | map00380 | Tryptophan metabolism | 0.48027715 | 0.897618646 | 3 |
|  | map03015 | mRNA surveillance pathway | 0.4930671 | 0.905992266 | 6 |
|  | map00730 | Thiamine metabolism | 0.50012789 | 0.905992266 | 1 |
|  | map00510 | N-Glycan biosynthesis | 0.50769379 | 0.905992266 | 3 |
|  | map04141 | Protein processing in endoplasmic reticulum | 0.52112888 | 0.905992266 | 16 |
|  | map03020 | RNA polymerase | 0.52200163 | 0.905992266 | 1 |
|  | map00130 | Ubiquinone and other terpenoid-quinone biosynthesis | 0.52554674 | 0.905992266 | 1 |
|  | map00051 | Fructose and mannose metabolism | 0.52956196 | 0.905992266 | 4 |
|  | map00280 | Valine, leucine and isoleucine degradation | 0.53762178 | 0.905992266 | 4 |
|  | map04122 | Sulfur relay system | 0.57627629 | 0.922930713 | 1 |
|  | map00410 | beta-Alanine metabolism | 0.58195085 | 0.922930713 | 4 |
|  | map00900 | Terpenoid backbone biosynthesis | 0.58746527 | 0.922930713 | 2 |
|  | map00531 | Glycosaminoglycan degradation | 0.59280996 | 0.922930713 | 2 |
|  | map00511 | Other glycan degradation | 0.59500295 | 0.922930713 | 4 |
|  | map00940 | Phenylpropanoid biosynthesis | 0.6094747 | 0.926156541 | 7 |
|  | map04712 | Circadian rhythm - plant | 0.61275389 | 0.926156541 | 8 |
|  | map00562 | Inositol phosphate metabolism | 0.63481788 | 0.952226821 | 3 |
|  | map00760 | Nicotinate and nicotinamide metabolism | 0.65765982 | 0.961503237 | 2 |
|  | map00513 | Various types of N-glycan biosynthesis | 0.70380735 | 0.976023578 | 2 |
|  | map00563 | Glycosylphosphatidylinositol (GPI)-anchor biosynthesis | 0.71285758 | 0.976023578 | 2 |
|  | map04120 | Ubiquitin mediated proteolysis | 0.72535679 | 0.976023578 | 10 |
|  | map03060 | Protein export | 0.72950485 | 0.976023578 | 1 |
|  | map00906 | Carotenoid biosynthesis | 0.73200566 | 0.976023578 | 1 |
|  | map00400 | Phenylalanine, tyrosine and tryptophan biosynthesis | 0.73648666 | 0.976023578 | 3 |
|  | map04130 | SNARE interactions in vesicular transport | 0.73648666 | 0.976023578 | 3 |
|  | map00360 | Phenylalanine metabolism | 0.77083533 | 0.999364856 | 3 |
|  |  |  |  |  |  |

| **Supplementary Table 24. GO annotation of genes up-regulated in aerial seeds. Related to Figure 5.** | | | | | | | | | |
| --- | --- | --- | --- | --- | --- | --- | --- | --- | --- |
| **GO ID** | **GO Term** | **GO Class** | **P-value** | **Adjusted P-value** | **Gene Number** | **Gene name** | **a_ch_s^+^** | **a_cl_s^+^** | **s_cl_s^+^** |
| GO:0006952 | defense response | BP | 0.0009 | 0.2224 | 5 | Aedg2G02054 | 68980.89 | 84648.03 | 48.00 |
|  |  |  |  |  |  | Aedg2G02055 | 1976.64 | 1518.05 | 4.57 |
|  |  |  |  |  |  | Aedg8G01513 | 1.81 | 1.42 | 0.00 |
|  |  |  |  |  |  | Aedg8G01377 | 1.08 | 1.26 | 0.00 |
|  |  |  |  |  |  | Aedg11G00071 | 37.23 | 18.60 | 0.11 |
| GO:0009870 | defense response signaling pathway | BP | 0.0208 | 0.2344 | 1 | Aedg11G00071 | 37.23 | 18.60 | 0.11 |
| GO:0006950 | response to stress | BP | 0.0321 | 0.2728 | 7 | Aedg2G02054 | 68980.89 | 84648.03 | 48.00 |
|  |  |  |  |  |  | Aedg2G02055 | 1976.64 | 1518.05 | 4.57 |
|  |  |  |  |  |  | Aedg11G00071 | 37.23 | 18.60 | 0.11 |
|  |  |  |  |  |  | Aedg9G00164 | 9.97 | 5.90 | 0.00 |
|  |  |  |  |  |  | Aedg8G01513 | 1.81 | 1.42 | 0.00 |
|  |  |  |  |  |  | Aedg5G00644 | 1.06 | 4.89 | 0.00 |
|  |  |  |  |  |  | Aedg8G01377 | 1.08 | 1.26 | 0.00 |
| GO:0042742 | defense response to bacterium | BP | 0.0479 | 0.3112 | 1 | Aedg11G00071 | 37.23 | 18.60 | 0.11 |
| GO:0050896 | response to stimulus | BP | 0.5356 | 0.7439 | 8 | Aedg2G02054 | 68980.89 | 84648.03 | 48.00 |
|  |  |  |  |  |  | Aedg2G02055 | 1976.64 | 1518.05 | 4.57 |
|  |  |  |  |  |  | Aedg11G00071 | 37.23 | 18.60 | 0.11 |
|  |  |  |  |  |  | Aedg9G00164 | 9.97 | 5.90 | 0.00 |
|  |  |  |  |  |  | Aedg9G00154 | 2.97 | 4.26 | 0.08 |
|  |  |  |  |  |  | Aedg5G00644 | 1.06 | 4.89 | 0.00 |
|  |  |  |  |  |  | Aedg8G01377 | 1.08 | 1.26 | 0.00 |
|  |  |  |  |  |  | Aedg8G01513 | 1.81 | 1.42 | 0.00 |
| + The table shows the relative FPKM of genes in the 3 types of seeds. | | | | | | | | | |

| **Supplementary Table 25. GO annotation of genes up-regulated in subterranean seeds. Related to Figure 5.** | | | | | | |  |  |  |
| --- | --- | --- | --- | --- | --- | --- | --- | --- | --- |
| **GO ID** | **GO Term** | **GO Class** | **P-value** | **Adjusted P-value** | **Gene Number** | **Gene name** | **a_ch_s^+^** | **a_cl_s^+^** | **s_cl_s^+^** |
| GO:0009247 | glycolipid biosynthetic process | BP | 0.0090 | 0.4017 | 3 | Aedg5G01092 | 0.05 | 1.59 | 3.76 |
|  |  |  |  |  |  | AedgUnG01597 | 0.04 | 0.08 | 1.01 |
|  |  |  |  |  |  | AedgUnG00036 | 0.00 | 0.23 | 0.37 |
| GO:0044262 | cellular carbohydrate metabolic process | BP | 0.3835 | 0.6793 | 2 | Aedg2G01057 | 2.79 | 1.20 | 6.82 |
|  |  |  |  |  |  | Aedg5G02460 | 0.02 | 0.01 | 5.14 |
| GO:0006629 | lipid metabolic process | BP | 0.6073 | 0.8552 | 5 | Aedg9G00493 | 0.50 | 0.54 | 29.79 |
|  |  |  |  |  |  | Aedg7G01304 | 8.10 | 3.05 | 12.99 |
|  |  |  |  |  |  | Aedg2G01057 | 2.79 | 1.20 | 6.82 |
|  |  |  |  |  |  | AedgUnG00005 | 0.29 | 0.32 | 3.80 |
|  |  |  |  |  |  | Aedg3G01079 | 0.20 | 0.18 | 1.73 |
| GO:0008610 | lipid biosynthetic process | BP | 0.6639 | 0.8858 | 2 | Aedg2G01057 | 2.79 | 1.20 | 6.82 |
|  |  |  |  |  |  | AedgUnG00005 | 0.29 | 0.32 | 3.80 |
| GO:0055114 | oxidation-reduction process | BP | 0.7764 | 0.9784 | 21 | Aedg11G02512 | 189.23 | 856.58 | 1622.22 |
|  |  |  |  |  |  | Aedg10G00791 | 1.22 | 0.93 | 120.00 |
|  |  |  |  |  |  | Aedg10G01411 | 0.00 | 41.17 | 112.60 |
|  |  |  |  |  |  | Aedg2G00562 | 1.86 | 0.62 | 52.06 |
|  |  |  |  |  |  | AedgUnG01048 | 1.35 | 0.13 | 47.56 |
|  |  |  |  |  |  | Aedg9G00004 | 1.91 | 1.19 | 39.48 |
|  |  |  |  |  |  | Aedg2G02384 | 2.58 | 4.99 | 28.00 |
|  |  |  |  |  |  | Aedg6G01550 | 2.91 | 3.60 | 17.48 |
|  |  |  |  |  |  | AedgUnG00376 | 1.02 | 0.14 | 12.03 |
|  |  |  |  |  |  | Aedg1G01185 | 0.46 | 0.68 | 7.26 |
|  |  |  |  |  |  | Aedg7G01630 | 0.92 | 3.18 | 5.11 |
|  |  |  |  |  |  | Aedg5G02531 | 0.22 | 1.09 | 3.32 |
|  |  |  |  |  |  | Aedg9G00469 | 0.00 | 0.00 | 3.27 |
|  |  |  |  |  |  | Aedg3G00746 | 0.05 | 0.29 | 3.22 |
|  |  |  |  |  |  | Aedg5G00510 | 0.13 | 0.02 | 3.19 |
|  |  |  |  |  |  | Aedg11G00098 | 0.00 | 1.02 | 2.16 |
|  |  |  |  |  |  | Aedg8G02074 | 0.03 | 0.00 | 1.60 |
|  |  |  |  |  |  | Aedg10G01410 | 0.00 | 3.61 | 0.95 |
|  |  |  |  |  |  | Aedg2G02370 | 0.01 | 0.05 | 0.91 |
|  |  |  |  |  |  | AedgUnG01210 | 0.01 | 0.00 | 0.79 |
|  |  |  |  |  |  | Aedg11G00093 | 0.00 | 0.68 | 0.72 |
| + The table shows the relative FPKM of genes in the 3 types of seeds. | | | | | | | | | |
